# A discrete model for the evaluation of public policies: The case of Colombia during the COVID-19 pandemic

**DOI:** 10.1371/journal.pone.0275546

**Published:** 2023-02-14

**Authors:** Alexandra Catano-Lopez, Daniel Rojas-Diaz, Diana Paola Lizarralde-Bejarano, María Eugenia Puerta Yepes

**Affiliations:** Department of Mathematical Sciences, Universidad EAFIT, Medellín, Colombia; Texas A&M University College Station, UNITED STATES

## Abstract

In mathematical epidemiology, it is usual to implement compartmental models to study the transmission of diseases, allowing comprehension of the outbreak dynamics. Thus, it is necessary to identify the natural history of the disease and to establish promissory relations between the structure of a mathematical model, as well as its parameters, with control-related strategies (real interventions) and relevant socio-cultural behaviors. However, we identified gaps between the model creation and its implementation for the use of decision-makers for policy design. We aim to cover these gaps by proposing a discrete mathematical model with parameters having intuitive meaning to be implemented to help decision-makers in control policy design. The model considers novel contagion probabilities, quarantine, and diffusion processes to represent the recovery and mortality dynamics. We applied mathematical model for COVID-19 to Colombia and some of its localities; moreover, the model structure could be adapted for other diseases. Subsequently, we implemented it on a web platform (MathCOVID) for the usage of decision-makers to simulate the effect of policies such as lock-downs, social distancing, identification in the contagion network, and connectivity among populations. Furthermore, it was possible to assess the effects of migration and vaccination strategies as time-dependent inputs. Finally, the platform was capable of simulating the effects of applying one or more policies simultaneously.

## 1 Introduction

Mathematical models are versatile tools for the study of epidemiological events. Such models can describe the transmission and control of pandemical infectious diseases, such as influenza and SARS [1, 2]. In recent years, researchers have stressed the important of modeling diseases considering their natural history or the social dynamics of the population, e.g., the spatial heterogeneity of the population, the incubation and latency periods, the sources of contagion, and the recovery process [3, 4]. Thus, the use of mathematical models is widespread, especially in designing and evaluating control policies [5, 6].

The COVID-19 pandemic was a turning point in the use of mathematical models for design or evaluating public policies, because its impact reshaped society dynamics and created the necessity of new public policies [7, 8]. The models have been implemented for planning critical gaps to reduce the effect of a pandemic and identify long- and short-term impacts caused by the disease [7]. Forecasting models could be classified as statistical, machine learning-based, and mechanistic. Some researchers implemented the mechanistic to ease the monitoring of an epidemic, because they can calibrate them using disease incidence data seen until a day and then simulate the model forward in time to produce the future time series [7, 9–11].

We identified several attempts to propose and apply models with mathematical expressions. These includes classical compartmental models [12] alongside Erlang models [13], integrodifferential equations to simulate infection process [14, 15], delay-differential equations to include latency period [16], partial differential equations for spatial heterogeneity [17, 18], and meta-populations for age-directed control strategies [19]. Also, we found that there are several mathematical models formulated in multiple countries to understand the complex transmission pattern of the COVID-19 pandemic [12]. However, the solution to complex mathematical expressions, as mentioned above, involves complex numerical methods or the acceptance of some assumptions to simplify other dynamics within the model [20]. Moreover, the intuition behind those mathematical expressions usually came from discrete-time approaches, and numerical methods proposed to solve them rely on time-domain discretization [20].

From the previous observations, we can point out that there is an opportunity to develop mathematical models that include and provide more information about the social and biological dynamics related to the disease, i.e., push the modeling towards more complex but realistic scenarios. Such information makes up a fundamental input for decision-makers when developing control strategies since it would make possible to identify population behaviors that influence the progress of the disease. Hence, in this study, we formulated and designed a discrete compartmental model with diffusion systems based on the natural history of the disease to simulate the effects of public health policies by defining parameters in correspondence with social behaviors, e.g., lock-downs, case-tracking programs, among others. Furthermore, it is possible to parameterize time series to simulate migration processes and vaccination programs as model inputs. We highlight that we can extrapolate the model to other diseases by assembling the model components considering the features of the disease.

The paper is divided as follows. The methodology section in which we describe the natural history of the disease, the basic model structure and its assumptions, the process of parameter estimation, and its validation. Then, in the results section, we present the model structure and its description; the model fitting for six localities, and the simulation of different public policies to control the COVID-19 spread in Colombia. Finally, we highlight that the notation will be capital letters for functions and lower case for parameters. Also, to simplify the notation, we will define all time-dependent states without (*t*) through the explanations document and, write functions that depend on multiple estates, we will abbreviate it as (⋅).

## 2 Materials and methods

### 2.1 Disease epidemiological description

COVID-19 is a respiratory syndrome generated by the SARS-CoV-2 virus and isolated for the first time in the Chinese city of Wuhan at the end of 2019 [21]. There are three know-routes of COVID-19 infection: (i) direct contact with infectious individuals, (ii) inhalation of micro-drops in the environment emitted by people carrying the virus, and (iii) contact with surfaces contaminated with the virus [22–24]. COVID-19 has latency and incubation periods, i.e., some individuals are presymptomatic and might be contagious before developing symptoms [25]. Additionally, some of them could be asymptomatic and still transmit the disease.

The detection of the asymptomatic population relies on two key strategies: (i) performing intensive COVID-19 tests over the population and (ii) identifying individuals who came into close contact with an infected person (contagion network) [26]). Despite the high saturation that COVID-19 has generated in health systems worldwide, the affected localities carry out reliable daily records based on clinical data to infer the disease trend through clinical data. Generally, such data comprises the positive, the death, and the recovered individuals.

### 2.2 Model assumptions and mathematical structure

We searched in the literature for a model that could serve as a basic structure. We chose the one proposed by [27] because it comprises the *SEIR* flow (susceptible, exposed, infected, and recovered) besides an environmental reservoir *T*. Due to the nature of the data (daily cases and death reports), we followed the advice in [2] to manage the modeling problem as a discrete-type one. Also, to overcome homogeneous population assumption and include the social behavior in infection transmission, we included contagion probabilities, implemented a free-circulation and quarantine, and a diffusion system during the viral infection.

#### 2.2.1 Free circulation, quarantine, and detection as components of contagion

**Fig 1:**
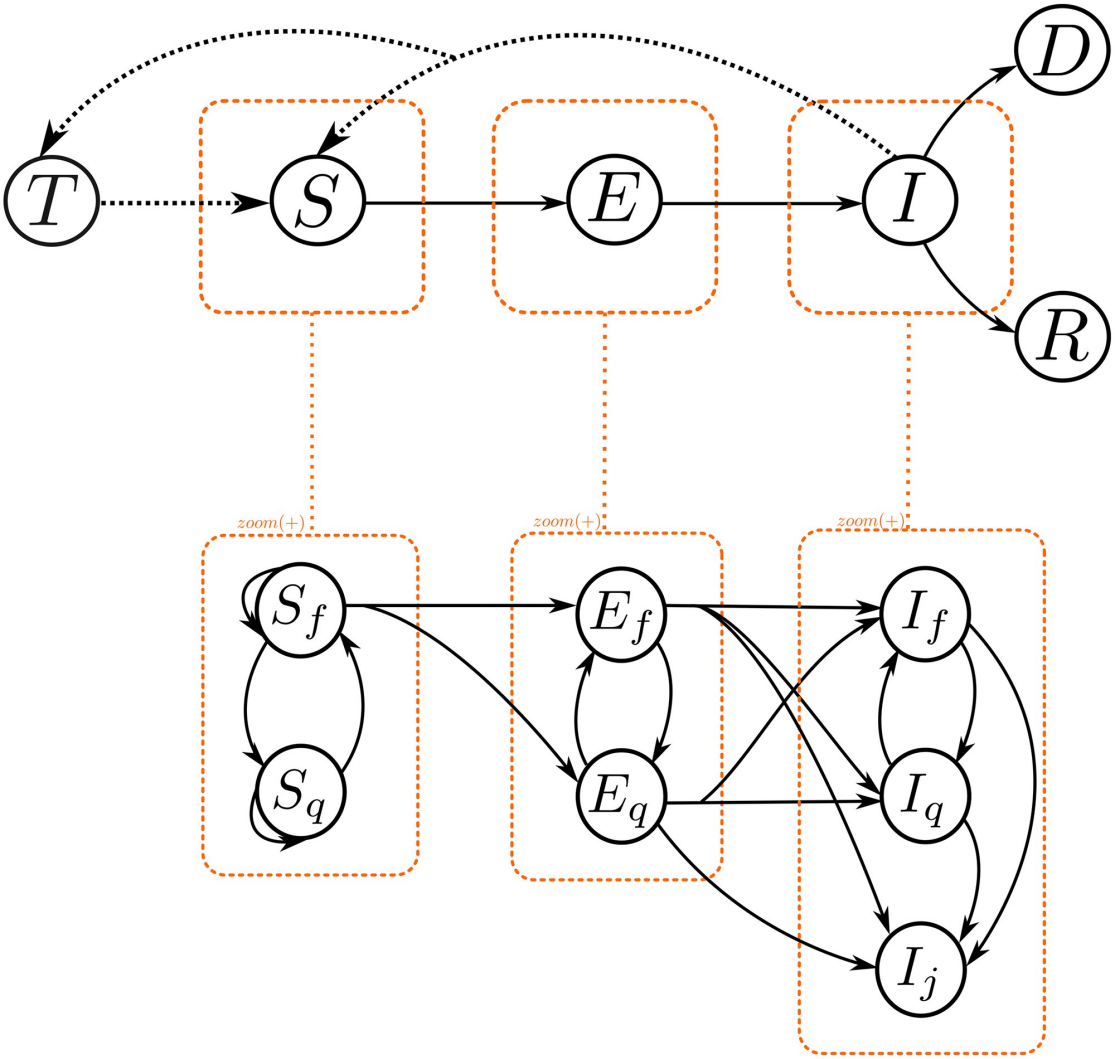
Compartmental diagram for a *SEIRD* model with *f* ⇌ *q*|*j* and infection process from environmental reservoir *T*. This include exposed (*E*) and death (*D*) compartments.

We treat quarantine (*q*) and free circulation (*f*) as a flow that affects the total population: the individuals in free circulation could enter quarantine and vice versa. We define *q* (quarantined) as the sub-index to indicate isolation and *f* (free) as the sub-index to indicate free circulation. Now, *S*_*q*_ will denote the isolated susceptible populations, *S*_*f*_ will denote those that remain in free circulation, and so on for other compartments as *I*. We refer to the flow between *f* and *q* as *f* ⇌ *q*. Isolation is relevant for the susceptible and infected since the lack of interactions protects them against contagion and isolated carriers can not spread the disease. Every compartment that belongs to the epidemic flow (e.g., *SIR*, *SEIR*, *SIS*) also allows for a partition into individuals of *q* and *f* kind (see Fig 1).

We define the flow state compartments as probabilities: λ_*qf*_ is the probability of change from state *q* to *f* for the next time step, and λ_*fq*_ the probability of the other flow direction. The population staying in *q* (e.g. *S*_*q*_ or *I*_*q*_) at time *t* + 1 only depends on λ_*qf*_, since infectious interactions are possible only for individuals in *f* (e.g. *S*_*f*_ and *I*_*f*_). Finally, we propose an extra flow, the infected detection *j*, which is the process of carrier identification (e.g. *I*_*j*_), which can also be treated as a variation of the *q* (*f* ⇌ *q*|*j* flow). Thus, *j* behaves as an absorbant state as long as the individual recovers (*R*).

For free circulating populations, it is essential to formulate a function that describes the probability that a susceptible contracts the disease. Thus, we implemented novel contagion functions proposed in [28], as the reader can consult in section 3.1.1.

#### 2.2.2 Diffusion systems in transmission models

Some authors consider that the time elapsed from the moment an individual enters a compartment is a determinant for dynamics such as infectious capacity (pathogen load) or recovery from disease [29]. Some mathematical expressions that include such information during modeling are integrodifferential terms or large chains of coupled compartments with different output rates (Erlang models). However, both approaches are equivalent in certain epidemiological circumstances, as pointed out in [30]. Thus, we propose a discrete analog to the Erlang model approach, considering the *f* ⇌ *q*|*j* flow.

In Fig 1, we show how a *SEIRD* model turns into a directed graph where each node has an associated population at every time step. Individuals in node can move to another node on the next time-step, provided an edge connecting both nodes. Such displacements obey probabilities, but we treat them as proportions, the discrete equivalent of rates. We can model some of the flows between compartments as random walks, i.e., through a matrix approach. We called the set of compartments that admit random walk as a *diffusion system*. Thus, it is possible to represent the entire state as a vector where each position corresponds to a node. To estimate these proportions we introduced to the model a parameter related to scaling (*γ*) and two parameters related to the shape of a beta distribution function (*a*, *b*), as described in detail in section 3.1.2.

#### 2.2.3 Mathematical and biological considerations during modeling

To formulate the model, we considered the social and biological behavior of the spread of COVID-19 over a population. However, we limited the scope by stating some assumptions which we summarize in the 15 items in Box 1.

**Assumptions for COVID-19 modeling**

1. The spread of the disease occurs only between people freely circulating (*f*). Thus, isolated carriers do not spread the disease (*j*, *q*).
2. The number of interactions that an individual can establish decreases when the proportion of individuals in isolation (*q* and *j*) rises.
3. The presymptomatic individuals can spread the disease.
4. Presymptomatic transmitters have lesser infectious capacity than symptomatic ones.
5. Infected people can develop severe or mild symptoms.
6. Individuals that develop mild symptoms are indistinguishable from asymptomatic ones.
7. Individuals that develop severe symptoms are immediately detected (or isolated and then detected).
8. Only people who develop severe symptoms can die.
9. Probabilities of recovery and death are functions of the number of days since developing symptoms.
10. We do not consider other death causes than COVID-19.
11. Recovered individuals do not lose immunity in short periods.
12. The population under study has no interactions with external populations.
13. The population is not homogeneous, instead, it could be represented as clusters of densely connected individuals.
14. There are only two infectious sources: living carriers and the environmental reservoir. All the infection probabilities are independent with each other.
15. Population renewal is negligible for short periods.

### 2.3 Parameter estimation, validation and methods for parameters variation

We took COVID-19 data for several localities in Colombia and periodically updated them by fitting the proposed model. Also, for each locality, we perform parameter estimation and validation process. Then, we implemented it on a web platform to simulate different control scenarios. In this paper, we present the fitting results for six localities: (i) the whole country, Colombia, with 50,372,424 inhabitants; (ii) the three largest cities in the country, Bogotá, Medellín, and Cali with 7,743,955, 2,533,424 and 2,252,616 inhabitants, respectively; (iii) the largest port city Barranquilla, with 1,274,250 inhabitants, and (iv) a city on the border with Brazil and Perú, Leticia, with 49,737 inhabitants.

We fit the model to data available from March 2020 to February 2022 in Datos Abiertos, a web portal where the Colombian government shares the registered data as an open resource. Thus, we fit the model for an ongoing pandemic considering the parameter variation over time, since some social parameters may vary between localities and periods. We divide the time series of the real data into some fragments to deal with abrupt changes (lock-downs, celebrations, among others), calling each fragment an *extension*. All the parameter estimations of the model for each locality were achieved through multiple extensions. This process depends on the socio-economic behavior in each locality. We illustrated this methodology in section 3.3.1 and S1 Appendix.

For each extension, we perform parameter estimations and validation processes using the final values of the previous extension as initial values of the subsequent extension. We performed parameter estimation by fitting the model to experimental data several times, using the following daily time series: the number of (i) current active cases (not accumulated), *J*, (ii) accumulated recovered cases, *R*_*j*_, and (iii) accumulated deaths, *D*. As the optimization algorithm we chose the inner point approach implemented in *GSUA_CSB* *Toolbox*, available in [31], under the *fmincon* function in Matlab2021a. The reader can consult linear restrictions for the optimization process coming from parameter intervals in Table 1. We used a cost function to measure the goodness of fit based on mean squared error and the Pearson correlation coefficient, also implemented in *GSUA_CSB* [31].

**Table 1:** Initial state and parameter definitions with their reference intervals. For further information about estimation intervals see [35–39].

**States**	**Definition**	**Reference interval**	**Units**
*S*	Susceptible	-	H
*E*	Exposed	1000 ± 1000	H
*P*	Presymptomatic	100 ± 100	H
*L*	Low symptomatic	-	H
*H*	High symptomatic	-	H
*J*	Active detected carriers	-	H
*L*_*j*_	Low detected	-	H
*H*_*j*_	High detected	-	H
*D*	Dead	-	H
*R*	Recovered	-	H
*R*_*J*_	Identified recovered	-	H
*T*	Environmental reservoir	-	[virus]
**Params**.	**Definition**	**Reference interval**	**Units**
*β*_*L*_	Probability of developing the disease (*L* to *S*)	0.8 ± 0.1	-
*β*_*P*_	Probability of developing the disease (*P* to *S*)	0.4 ± 0.2	-
*β*_*T*_	Probability of developing the disease (*T* to *S*)	0.5 ± 0.5	-
λ_*fq*_	Probability of entry into quarantine	0.5 ± 0.5	-
λ_*qf*_	Probability of getting out of quarantine	0.5 ± 0.5	-
*ϑ*_*E*_	Effectiveness of the sanitary barrier	0.5 ± 0.5	-
*ϑ*_*P*_	Effectiveness in detecting *P*	0.5 ± 0.5	-
*μ*	Mortality scale parameter	0.5 ± 0.5	-
*γ*_*L*_	Recovery scale parameter for *L*	0.5 ± 0.5	-
*γ*_*H*_	Recovery scale parameter for *H*	1.5 ± 1.5	-
*δ*	Probability of being a member of *H*	0.075 ± 0.075	-
*ϕ*_*EP*_	Rate referring to the latency period	4 ± 4	*t* ^−1^
*ϕ*_*PL*_	Rate referring to the incubation period of *L*	1.5 ± 1.5	*t* ^−1^
*ϕ*_*PH*_	Rate referring to the incubation period of *H*	1.5 ± 1.5	*t* ^−1^
*ϕ*_*T*_	Virus removal rate from the environment	4.5 ± 3.5	*t* ^−1^
*η*_*L*_	Symptomatic behavior modifier	0.5 ± 0.5	-
*η*_*ϑ*_	*ϑ*_*P*_ modifier for the *L* case	0.5 ± 0.5	-
*k*_*L*_	*L* contribution to the virus reservoir	5.5 ± 4.5	[virus](*tH*)^−1^
*k*_*P*_	*P* contribution to the virus reservoir	1 ± 0	[virus](*tH*)^−1^
*a*_*L*_	First *L* recovery distribution parameter	8 ± 7	-
*b*_*L*_	Second *L* recovery distribution parameter	8 ± 7	-
*a*_*H*_	First *H* recovery distribution parameter	8 ± 7	-
*b*_*H*_	Second *H* recovery distribution parameter	8 ± 7	-
*a*_*μ*_	First *H* mortality distribution parameter	8 ± 7	-
*b*_*μ*_	Second *H* mortality distribution parameter	8 ± 7	-
*z*	Mean of interactions of an individual per day	15 ± 15	(*Ht*)^−1^
*ν*	Connection parameter	250 ± 250	-
m	Maximum duration of symptoms	42.5 ± 17.5	t

For model validation, we implemented different methodologies such as sensitivity (SA), uncertainty (UA), and practical identifiability (IA) analyses, as described in [32]. We described the algorithm proposed in S1 Appendix for parameter estimation and validation for each extension. All the implemented routines and methodologies, are available in a GitHub repository 1 [33].

## 3 Results

We divide this section into three major results: first, we describe mathematical preliminaries of contagion probabilities, diffusion systems, their corresponding compartments, and how they merge into a compartmental model. Then, we present the model fitting for different affected localities in Colombia. Finally, we describe the implementation of the model on a web platform to evaluate the effects of public policies to control the diseases in some localities of Colombia.

### 3.1 Preliminaries: Mathematical structure for COVID-19 model

The proposed model involves: (i) multiple quarantine and free circulation flow, (ii) contagion probabilities, and (iii) diffusion systems. For more information and details, we describe the complete mathematical expressions and their corresponding dynamics in section 3.2. First, we will describe the left-right compartment flow of the model as shown in the orange rectangles in Fig 1.

The first flow goes from *S* to *E*, and the compartment *E* is divided into two levels to include a delay in the exposed state. In this way, the latency period is at least two days, and only individuals in *E*^2^ can become infectious at time *t* + 1 (where the super-index represents the infection day). Then, following assumptions 3, 5, and 6, we split *I* into three different states: (i) presymptomatic *P*, constituted by those individuals who have no symptoms but can transmit the disease; (ii) low-symptomatic *L*, constituted by those individuals who developed mild symptoms after the incubation period had passed; and (iii) severe or high symptomatic *H* constituted by those individuals who developed severe symptoms after finishing the incubation period.

We include the possibility of becoming low or high symptomatic during the presymptomatic step. Thus, we split the flow from *E*^2^ to *P*, into *P*^*H*^ and *P*^*L*^; where the super-script in the *P* case suggests the severity of the symptoms that individuals will develop (low and high). Then, we define flows from *P*^*L*^ to *L* and *P*^*H*^ to *H*, respectively. Finally, when the population becomes *H* or *L*, they enter the diffusion systems described in section 2.2.2. Even so, note that only *H* case has a probability of death and both, *L* and *H*, have a probability of recovery.

We can define different sets of populations inside the model: the total alive population *N* = *S* + *E* + *I* + *R*, with the total population of susceptible *S* = *S*_*f*_ + *S*_*q*_; total exposed individuals $E=E_{f}^{1}+E_{q}^{1}+E_{f}^{2}+E_{q}^{2}$. Total infected population *I* = *P* + *L* + *H*, and the total presymptomatics $P=P_{f}^{L}+P_{q}^{L}+P_{j}^{L}+P_{f}^{H}+P_{q}^{H}+P_{j}^{H}.$ Finally, we define *H* and *L* as all the individuals belonging to those diffusion systems described in section 3.1.2.

#### 3.1.1 Contagion probabilities overcoming homogeneous assumption

From assumptions 1, 3, and 4, it follows that infectious interactions that involve *P*_*f*_ are less likely to result in new infections than the interactions involving *L*_*f*_. We define the probability of infection after infectious interaction with presymptomatic, *β*_*P*_, and after interaction with symptomatic, *β*_*L*_, treating each type of interaction as a different process. Considering assumption 2, a natural expression for the number of interactions that the infected individuals has with the susceptible population is *zI*_*f*_*S*_*f*_/*N*, being *z* the number of average interactions that a regular individual has by time-unit. In this way, the presymptomatic contact function would be given by

$$\begin{array}{c} \begin{array}{ccc} \Phi_{P}\left(S_{f}\left(t\right),P_{f}\left(t\right),I\left(t\right),N\left(t\right)\right) & = & 1-[1-\frac{1}{S_{f}\left(t\right)}{]}^{zP_{f}\left(t\right){\left[\frac{S_{f}\left(t\right)}{N(t)}\right]}^{ℵ(S_{f}\left(t\right),I\left(t\right))}}\\ ℵ(S_{f}\left(t\right),I_{f}\left(t\right)) & = & 1+\nu \frac{I_{f}\left(t\right)}{S_{f}\left(t\right)+I_{f}\left(t\right)} \end{array} \end{array}$$

where the expression ℵ(⋅) represents the heterogeneity of the population (ℵ(⋅) ≈ 0 is a homogeneous population), and it is introduced to decrease the probability of contact whenever *I*_*f*_ increases or *S*_*f*_ decreases. *ν* is the parameter of intrinsic isolation. We define the symptomatic contact function as

$$\Phi_{L}\left(S_{f}\left(t\right),L_{f}\left(t\right),I\left(t\right),N\left(t\right)\right)=1-[1-\frac{1}{S_{f}\left(t\right)}{]}^{zL_{f}\left(t\right){\left[\frac{S_{f}\left(t\right)}{N(t)}\right]}^{ℵ(S_{f}\left(t\right),I\left(t\right))}}.$$

Finally, to define the probability of infection from an environmental source, let *T*(*t*) represents the size of the reservoir at time *t* and set *k*_*L*_ as the mean contribution to the reservoir of a typical symptomatic carrier *L*(*t*) per unit of time. It would be impossible to avoid contact with the reservoir if all the individuals become infected. Then we assume that a reservoir size of *k*_*L*_*N*(*t*), where *N*(*t*) is the size of the whole population, represents an extreme scenario, i.e., that *T*(*t*) ≤ *k*_*L*_*N*(*t*). Now, assuming that *k*_*L*_*N*(*t*) ≈ *k*_*L*_*N*(0), we consider that the probability of having contact with the reservoir Φ_*T*_ must be equal to the numerical closeness between *T*(*t*) and *k*_*L*_*N*(0). So, we defined the contact function with the environmental source as

$$\Phi_{T}\left(T\left(t\right),S\left(t\right),I\left(t\right)\right)=\text{min}(1,\;[\frac{T(t)}{k_{L}N\left(t\right)}{]}^{ℵ(S_{f}\left(t\right),\;I_{f}\left(t\right))})$$

To achieve the related contagion functions, it only remains to multiply each contact function by its respective probability of infection (*β*_*P*_, *β*_*L*_, *β*_*T*_). From assumption 14, the probability of not being infected would be the multiplication of the complements of infection probabilities given by the contagion functions.

#### 3.1.2 Diffusion systems in transmission models with quarantine compartments

According to assumptions 7, 8, and 9, we divided the classical compartment *L* and *H* into time-since-event compartments, because we can represent them as flows, as in [15, 34]. First, we assumed to have a sampling time unit of one day and set a maximum number of days-since-event to model m within the selected compartment. Then we represented by sub-compartments (node with level) *L*^*τ*^ and *H*^*τ*^ where τ∈{0,...,m-1}, the population of individuals that remain infected after *τ*-days since they became infected. We refer to each sub-compartment as a node, each compartment as a state, where the super-indices (*τ*) as the *level* of the node (related to disease time development). On the other hand, sub-indices will indicate the *type* of the node (*f*, *q*, or *j*).

The advantage of introducing multiple levels for symptomatic states, resides in the fact that we can define the probability of recovery from disease, the probability of dying because of the disease, and the capacity of disease transmission, as functions of the number of days since symptoms development (similar to the case of integrodifferential terms for continuous-time case).

**Fig 2:**
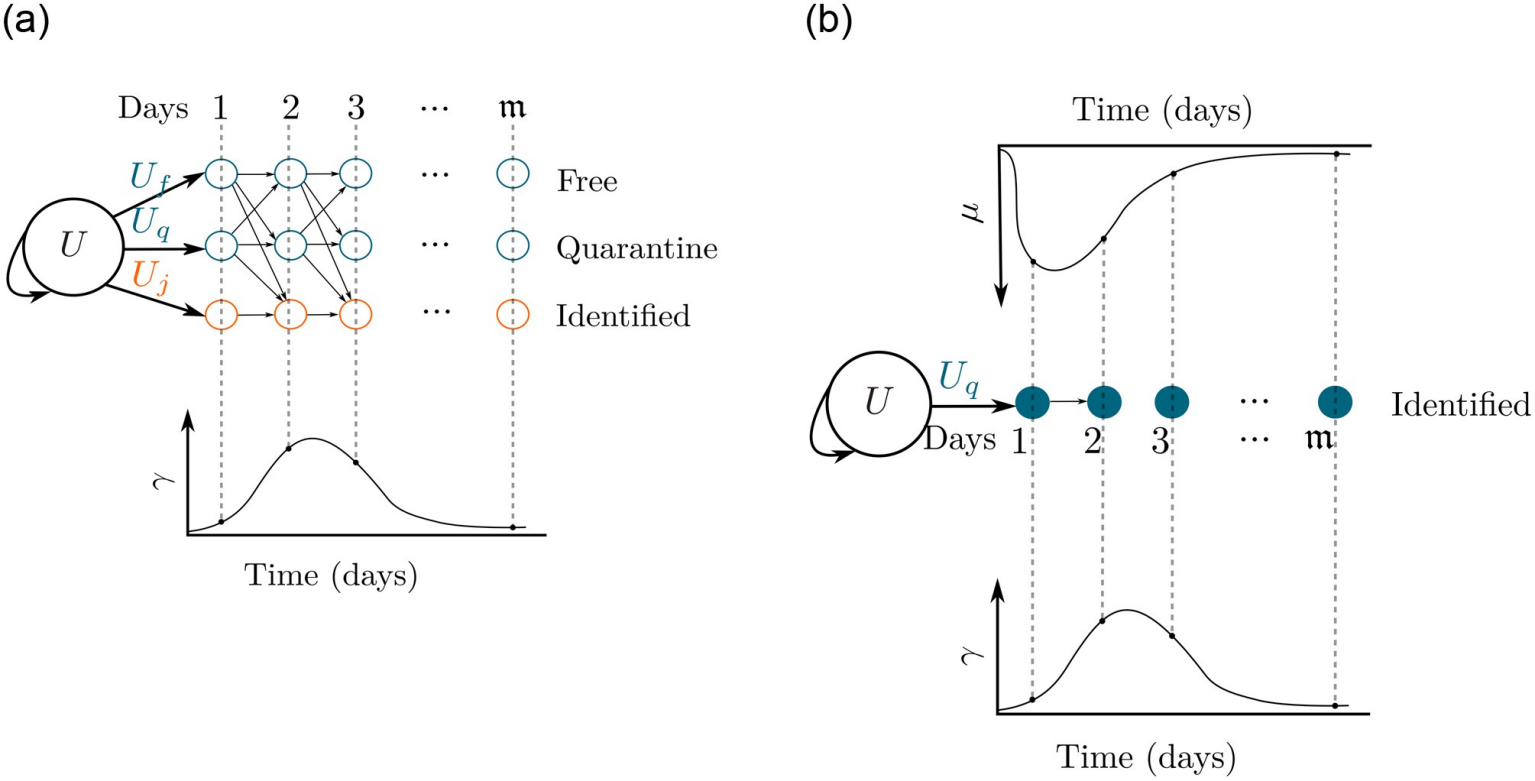
Example of two cases for diffusion systems. We propose discretized beta-shape functions to assign a probability for recovery or death in each level. Assigning an enumeration for the nodes and a priority for flows, it is possible to define correspondent random walk matrices.

However, in this paper, we focus on recovery and death functions. To estimate those functions we define three parameters: *a* and *b* as the shape parameters of a beta-distribution function, and *γ* ∈ [0, 1] as a parameter of scale. Then, we split the domain of the beta-distribution function into m equally spaced points to achieve a discrete function and normalize the values of such discrete functions to fit in the interval [0, *γ*], being 0 the lowest value of the discrete function and *γ* the highest value. Such beta-shaped function evaluated at each point will give the probability of recovery or death for the corresponding level (see Fig 2).

**Fig 3:**
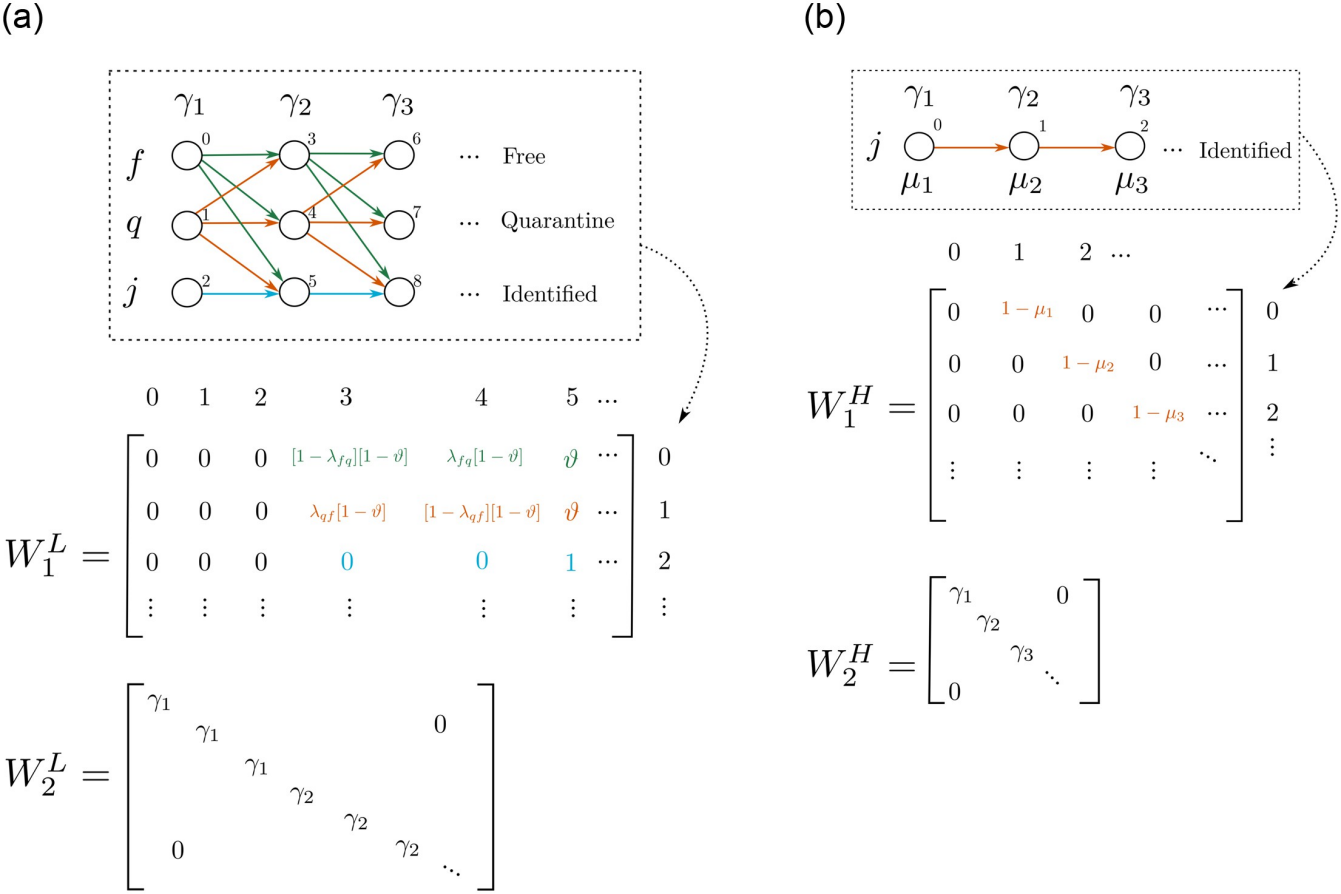
Example of diffusion matrices for *L* and *H* deduced from an order for the nodes (enumeration). Note that individuals in *L* have *f* ⇌ *q*|*j* flow (matrix $W_{1}^{L}$) and recover flow (matrix $W_{2}^{L}$). λ_*qf*_ and λ_*fq*_ are the probabilities of incoming or leaving quarantine, respectively; *ϑ* is the probability of being identified as infected. Individuals in *H* can recover (matrix $W_{2}^{H}$) or die (matrix $W_{1}^{H}$). Values for *γ*_*i*_ and *μ*_*i*_ are obtained as shown in Fig 2. For the mathematical description of the matrix, see sections 3.2.4 and 3.2.5. (a) Diffusion process for *L*, (b) Diffusion process for *H*.

We found in the literature that the virus could remain for 41 days or less for some reported cases [35]. Hence, we set m=41 and define each recovery function (for *L* and *H*) and death function (for *H*) as explained previously, i.e, through a parameterized and discrete beta-shaped function. In this way, we get three types and 41 levels for state *L*, for a total of 123 nodes, and a single type with 41 levels for *H* (41 nodes). Once the nodes have been established and numerated, it only remains to define random-walk matrices to easily model the flow inside *L* and *H* states. We enumerated the nodes as can be seen in Fig 3 and chose the following priority of events: recovery > dead > *f* ⇌ *q*|*j*. Priority means that an individual who recovers cannot die, and so on. Thus, we defined a matrix for each flow and multiply them to achieve the random walk matrix. For instance, the matrix $W_{1}^{L}$ in Fig 3 represents the *f* ⇌ *q*|*j* transversal flow; the matrix $W_{2}^{L}$ represents the process of recovery from disease for *L*; $W_{1}^{H}$ represents the process of dying from disease for *H*; and $W_{2}^{H}$ the process of recovery from the disease for *H*.

Finally, the random walk matrix for *L* is given by

$$\begin{array}{c} W^{L}=\left(I-W_{2}^{L}\right)W_{1}^{L} \end{array}$$ (1)

and the random walk matrix for *H* is given by

$$\begin{array}{c} W^{H}=\left(I-W_{2}^{H}\right)W_{1}^{H}. \end{array}$$ (2)

which is strictly triangular superior. In sections 3.2.4 and 3.2.5, we define mathematically the diffusion systems, which include a beta-distribution function to describe the probabilities to flow from one compartment to another one.

### 3.2 The mathematical models: COVID-19 case

**Fig 4:**
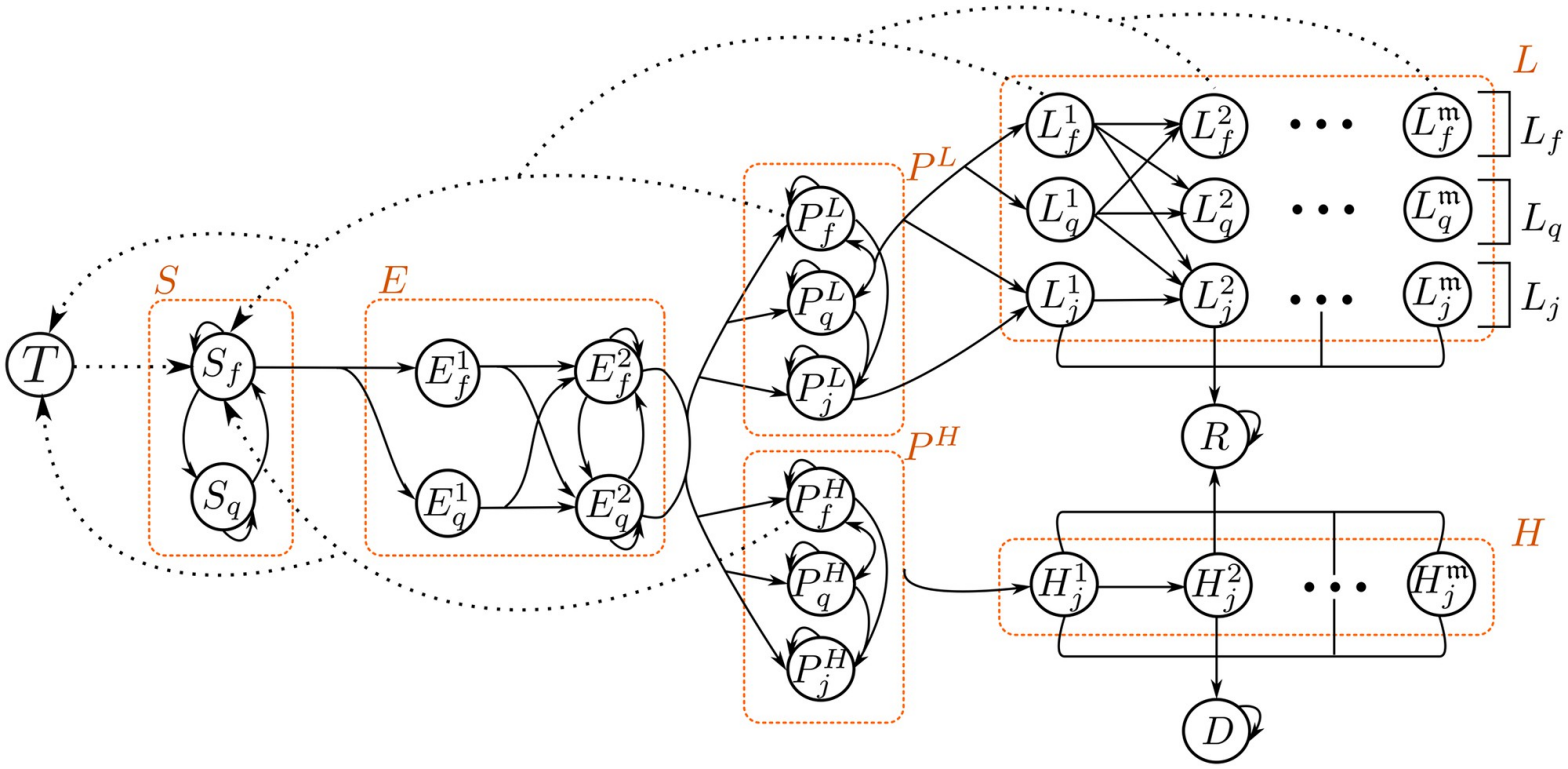
The model built from the basic structure proposed in section 2.2 and Fig 1. Note that both structures share the same compartments but extend some in sub-compartments such as quarantine, free circulation, and identified cases. We define the symptomatic compartments as diffusion systems, representing the recovery and death of the disease process. For further information about the diffusion systems, see section 3.1.2.

In this subsection, we describe the mathematical model presented in Fig 4, together with their mathematical expressions for each compartment and diffusion system. In Table 1, we describe the states and parameters implemented in the model with their feasible ranges and units.

#### 3.2.1 Susceptible state (S)

We divide the susceptible population into two sub-compartments: those in free circulation, *S*_*f*_, and those in voluntary quarantine, *S*_*q*_. Event probabilities and their complements define the flow between compartments. For example, the number *S*_*f*_ is given by the probability of not becoming infected from any infectious resource ((1 − Φ_*L*_(⋅)*β*_*L*_), (1 − Φ_*P*_(⋅)*β*_*P*_), (1 − Φ_*T*_(⋅)*β*_*T*_)), and do not entered into quarantine (1 − λ_*fq*_) plus those who left quarantine (λ_*qf*_). Similarly, *S*_*q*_ depends on the probability of entering quarantine due to not becoming infected and leaving quarantine. But, if there is an infectious interaction in *S*_*f*_, they become exposed and pass to the *E* compartment. The successive compartments follow the same idea of probabilities and their complement, even with more events, as we will see below.

$$\begin{array}{cc} S_{f}\left(t+1\right) & =\lambda_{qf}S_{q}\left(t\right)+\left(1-\lambda_{fq}\right)S_{f}\left(t\right)[1-\Phi_{P}\left(\cdot \right)\beta_{P}][1-\Phi_{L}\left(\cdot \right)\beta_{L}]\\ & \;[1-\Phi_{T}\left(\cdot \right)\beta_{T}]\\ S_{q}\left(t+1\right) & =\left(1-\lambda_{qf}\right)S_{q}\left(t\right)+\lambda_{fq}S_{f}\left(t\right)[1-\Phi_{P}\left(\cdot \right)\beta_{P}][1-\Phi_{L}\left(\cdot \right)\beta_{L}]\\ & \;[1-\Phi_{T}\left(\cdot \right)\beta_{T}] \end{array}$$ (3)

#### 3.2.2 Exposed state (E)

Exposed individuals could circulate freely (*E*_*f*_) or stay in quarantine (*E*_*q*_) with probabilities λ_*fq*_ and λ_*qf*_; they have not developed enough viral load to be infectious. Some individuals can stay over two days in this subsystem because the flow from *E*^2^ to *P* is given by $e^{-\phi_{EP}}$. Those individuals who have already completed the latency period become *P*^*L*^ or *P*^*H*^.

$$\begin{array}{ccc} E_{f}^{1}(t+1) & = & (1-\lambda_{fq})S_{f}(t)[\Phi_{P}(\cdot )\beta_{P}+(1-\Phi_{P}(\cdot )\beta_{P})\Phi_{P}(\cdot )\beta_{L}\\ & & +(1-\Phi_{P}(\cdot )\beta_{P})(1-\Phi_{P}(\cdot )\beta_{L})\Phi_{P}(\cdot )\beta_{T}]\\ E_{f}^{2}(t+1) & = & \lambda_{qf}E_{q}^{1}(t)+[1-\lambda_{fq}]E_{f}^{1}(t)+\\ & & e^{-\phi_{EP}}[\lambda_{qf}E_{q}^{2}(t)+(1-\lambda_{f}q)E_{f}^{2}(t)]\\ E_{q}^{1}(t+1) & = & \lambda_{fq}S_{f}(t)[\Phi_{P}(\cdot )\beta_{P}+(1-\Phi_{P}(\cdot )\beta_{P})\Phi_{P}(\cdot )\beta_{L}\\ & & +(1-\Phi_{P}(\cdot )\beta_{P})(1-\Phi_{P}(\cdot )\beta_{L})\Phi_{P}(\cdot )\beta_{T}]\\ E_{q}^{2}(t+1) & = & \lambda_{fq}E_{f}^{1}(t)+[1-\lambda_{qf}]E_{q}^{1}(t)+e^{-\phi_{EP}}\\ & & \left[(1-\lambda_{qf})E_{q}^{2}(t)+\lambda_{fq}E_{f}^{2}(t)\right] \end{array}$$

#### 3.2.3 Low and high presymptomatic states (P)

*E* passes to *P* depending on the severity of the symptoms they develop. Note that individuals in presymptomatic compartments have enough viral load to infect a susceptible individual through direct contact, and they also contribute to the viral load in the environmental reservoir. The flow from *P* to *L* and *H* are defined by $e^{-\phi_{PL}}$ and $e^{-\phi_{PH}}$, respectively which represent probabilities of transitions between the compartments.

- **Low presymptomatic** (*P*^*L*^): we defined them as individuals that would develop mild symptoms or no symptoms. We assumed that the disease effect of this population does not cause death. Also, this compartment has sub-compartments that represent free-circulation, $P_{f}^{L}$ with probability λ_*qf*_, quarantine or self-isolation, $P_{q}^{L}$ with probability λ_*fq*_, and identified, $P_{j}^{L}$. The last one is a new sub-compartment, which causes obligatory isolation because the identification by the health system. We described it with probabilities *ϑ*_*E*_ (for those new presymptomatic) and *ϑ*_*P*_ (for those circulating presymptomatic). Finally, *δ* is the proportion of individuals that will develop severe symptoms and 1 − *δ* is its complement.
  $$\begin{array}{ccc} P_{f}^{L}(t+1) & = & \left[1-\delta ][1-\vartheta_{E}][1-e^{-\phi_{EP}}][(1-\lambda_{fq})E_{f}^{2}(t)+\lambda_{qf}E_{q}^{2}(t)\right]\\ & & +[1-\vartheta_{P}][e^{-\phi_{PL}}][(1-\lambda_{fq})P_{f}^{L}(t)+\lambda_{qf}P_{q}^{L}(t)]\\ P_{q}^{L}(t+1) & = & \left[1-\delta ][1-\vartheta_{E}][1-e^{-\phi_{EP}}][\lambda_{fq}E_{f}^{2}(t)+(1-\lambda_{qf})E_{q}^{2}(t)\right]\\ & & +[1-\vartheta_{P}][e^{-\phi_{PL}}][\lambda_{fq}P_{f}^{L}(t)+(1-\lambda_{qf})P_{q}^{L}(t)]\\ P_{j}^{L}(t+1) & = & \vartheta_{E}[1-\delta ][1-e^{-\phi_{EP}}][E_{q}^{2}(t)+E_{f}^{2}(t)]\\ & & +e^{-\phi_{PL}}\left[\vartheta_{P}[P_{f}^{L}(t)+P_{q}^{L}(t)]+P_{j}^{L}(t)\right] \end{array}$$
- **High presymptomatic** (*P*^*H*^): As in the *P*_*L*_ compartment, we divided this compartment into three sub-compartments as (i) free-circulation, $P_{f}^{H}$, (ii) quarantine, $P_{q}^{H}$, and (iii) identified, $P_{j}^{H}$; with similar probabilities as exposed in the *P*_*L*_ compartment case.
  $$\begin{array}{ccc} P_{f}^{H}(t+1) & = & \delta [1-\vartheta_{E}][1-e^{-\phi_{EP}}][(1-\lambda_{fq})E_{f}^{2}(t)+\lambda_{qf}E_{q}^{2}(t)]\\ & & +[1-\vartheta_{P}][e^{-\phi_{PH}(\cdot )}][(1-\lambda_{fq})P_{f}^{H}(t)+\lambda_{qf}P_{q}^{H}(t)]\\ P_{q}^{H}(t+1) & = & \delta [1-\vartheta_{E}][1-e^{-\phi_{EP}}][\lambda_{fq}E_{f}^{2}(t)+(1-\lambda_{qf})E_{q}^{2}(t)]\\ & & +[1-\vartheta_{P}][e^{-\phi_{PH}}][\lambda_{fq}P_{f}^{H}(t)+(1-\lambda_{qf})P_{q}^{H}(t)]\\ P_{j}^{H}(t+1) & = & \vartheta_{E}\delta [1-e^{-\phi_{EP}}][E_{q}^{2}(t)+E_{f}^{2}(t)]\\ & & +e^{-\phi_{PH}}\left[\vartheta_{P}[P_{f}^{H}(t)+P_{q}^{H}(t)]+P_{j}^{H}(t)\right] \end{array}$$

#### 3.2.4 Low symptomatic state (L)

Individuals in this compartment have sufficient viral load to transmit the disease and manifest mild symptoms or be asymptomatic. They will not die but will recover with some time-since-infection probability. Individuals in *L* can move between the free circulation and quarantine states or compartments. Also, they could be identified and isolated in *L*_*j*_. Low symptomatic carriers have a greater viral load than presymptomatic ones; thus, they contribute more to the environmental concentration of the virus.

We took into account some parameters related to COVID-19 control. First, we modified flows from *f* to *q* (λ_*fq*_) and from *q* to *f* (λ_*qf*_) for *L*, by defining a parameter *η*_*L*_ and implemented it as $\lambda_{fq}^{\eta_{L}}$ and $\lambda_{qf}^{1/\eta_{L}}$. Note that the higher the value for *η*_*L*_ the more similar the behavior of *L* and *P*. In contrast, when *η*_*L*_ → 0, the entire population in *L*_*f* + *q*_ ≔ *L*_*f*_ + *L*_*q*_ moves and remains in *L*_*q*_ unless they are detected. Also, we assumed the identification of presymptomatic individuals to be lesser than the identification of symptomatic ones. Hence, we proposed that $\vartheta_{P}^{\eta_{\vartheta }}$ percent (where *η*_*ϑ*_ ∈ [0, 1]) of the population in *L*_*f*+*q*_ is detected and moves into *L*_*j*_ for the next time step (whether they do not recover). Note that all the people in *P* that do develop severe symptoms are immediately detected as they flow into *H*_*j*_. Further, from assumption 7, the *L* has *f*, *q*, and *j* compartments, and the only compartment available for *H* is *j*.

As mentioned in section 3.1.2, we consider three types and 41 levels for the *L* state for a total of 123 nodes (or sub-states). Instead of writing down those 123 state-equations we defined a vector with 123 positions (one for each node) and a random walk matrix. However, it is quite difficult and unintuitive to present our results based on the position of that vector. So, we proceed as follow: let *ℓ*_1_(⋅) be a function that receives a node and returns its level, and *ℓ*_2_(⋅) a function that receives a node and returns its type. Also, let *N*_*s*_ denote the starting node for the flow, *N*_*a*_ the arrival node for that flow, and let *Q* the condition *ℓ*_1_(*N*_*a*_) = *ℓ*_1_(*N*_*s*_) + 1, for all *N*_*a*_ and *N*_*s*_ in the diffusion system. Flows within the diffusion system can be modeled through the function

$$W_{1}^{L}(N_{s},N_{a})=\left\{\begin{array}{ll} \left(1-{\left[\lambda_{fq}\right]}^{\eta_{L}})(1-\vartheta_{P}^{\eta_{\vartheta }}\right) & \text{if}\;(\ell_{2}(N_{s})=\ell_{2}(N_{a})=f)\;\text{and}\;Q\;\text{holds}\\ {\left[\lambda_{fq}\right]}^{\eta_{L}}(1-\vartheta_{P}^{\eta_{\vartheta }}) & \text{if}\;(\ell_{2}(N_{s})=f,\ell_{2}(N_{a})=q)\;\text{and}\;Q\;\text{holds}\\ \vartheta_{P}^{\eta_{\vartheta }} & \text{if}\;(\ell_{2}(N_{s})=f,\ell_{2}(N_{a})=j)\;\text{and}\;Q\;\text{holds}\\ {\left[\lambda_{qf}\right]}^{1/\eta_{L}}(1-\vartheta_{P}^{\eta_{\vartheta }}) & \text{if}\;(\ell_{2}(N_{s})=q,\ell_{2}(N_{a})=f)\;\text{and}\;Q\;\text{holds}\\ \left(1-{\left[\lambda_{qf}\right]}^{1/\eta_{L}})(1-\vartheta_{P}^{\eta_{\vartheta }}\right) & \text{if}\;(\ell_{2}(N_{s})=\ell_{2}(N_{a})=q)\;\text{and}\;Q\;\text{holds}\\ \vartheta_{P}^{\eta_{\vartheta }} & \text{if}\;(\ell_{2}(N_{s})=q,\ell_{2}(N_{a})=j)\;\text{and}\;Q\;\text{holds}\\ 1 & \text{if}\;(\ell_{2}(N_{s})=\ell_{2}(N_{a})=j)\;\text{and}\;Q\;\text{holds}\\ 0 & \text{otherwise} \end{array}\right.$$

while inside/out flows of *L* can be modeled by

$$\begin{array}{c} \begin{array}{ccc} W_{2}^{L}\left(N_{s}\right) & = & \gamma_{L}g_{\gamma }(\frac{\ell_{1}\left(N_{s}\right)}{m})/\underset{w\in \{1,\cdots ,m\}}{\text{max}}g_{\gamma }\left(\frac{w}{m}\right) \end{array} \end{array}$$

being the $W_{2}^{L}\left(N_{s}\right)$ the discrete beta-shaped function we mentioned in section 3.1.2 and *g*_*γ*_(*x*) a beta-distribution function parameterized by *a*_*L*_ and *b*_*L*_.

$$g_{\gamma }(x)=\left\{\begin{array}{cc} \frac{x^{a_{L}-1}{\left(1-x\right)}^{b_{L}-1}}{B(a_{L},b_{L})} & 0<x<1\\ 1 & 1\leq x\\ 0 & \text{otherwise} \end{array}\right.$$

It is worth to mention that the incoming flow of a node becomes the total content of the node for the next time step. That is because all individuals in time *t* change their level for time *t* + 1. For instance individuals in level *k* ≥ 1 and type *f* for time-step *t* + 1 would be given by

$$\begin{array}{ccc} L_{f}^{k}\left(t+1\right) & = & \sum_{w\in \{f,q,j\}}L_{w}^{k-1}\left(t\right)W_{1}^{L}\left(L_{w}^{k-1},L_{f}^{k}\right)\left(1-W_{2}^{L}\left(L_{w}^{k-1}\right)\right) \end{array}$$

where the sum does not consider any other levels than *k* − 1 because of the condition *Q* in function $W_{1}^{L}$. The expression $L_{w}^{k-1}\left(t\right)W_{1}^{L}\left(L_{w}^{k-1},L_{f}^{k}\right)$ in the multiplication above gives us the proportion of individuals in the starting node that will move into the arrival node for the next time-step following the f⇌q/j transversal flow. Multiplication by $\left(1-W_{2}^{L}\left(L_{w}^{k-1}\right)\right)$ allows us to only consider those individuals that do not recover. On the other hand, it is clear that

$$\begin{array}{c} \begin{array}{ccc} L(t) & = & \sum_{w\in \{f,j,q\}}\sum_{i=1}^{m}L_{w}^{i}\left(t\right) \end{array} \end{array}$$

It only remains to note that the first level for *L* would run empty for the next time-step unless we consider the income of new low-symptomatic individuals. Thus *L*^1^(*t* + 1) would be given by

$$\begin{array}{ccc} L_{f}^{1}(t+1) & = & \left[\left(1-\lambda_{fq})P_{f}^{L}(t)+\lambda_{qf}P_{q}^{L}(t\right)\right](1-\vartheta_{P})\\ L_{q}^{1}(t+1) & = & \left[\lambda_{fq}P_{f}^{L}(t)+(1-\lambda_{qf})P_{q}^{L}(t)\right](1-\vartheta_{P})\\ L_{j}^{1}(t+1) & = & P_{j}^{L}(t)+\vartheta_{P}(P_{f}^{L}(t)+P_{q}^{L}(t)) \end{array}$$

Note that we include three main strategies for the identification of the carriers of COVID-19: (i) tracing of contagion networks, i.e., the isolation of individuals who had suspected contact with an identified carrier; (ii) the identification of those presymptomatic carriers who eluded the first strategy. Finally, (iii) the isolation of those carriers that do exhibit symptoms. We model such strategies by including three parameters for the identification of infectious individuals. To comply with such assumptions, we proposed a parameter *ϑ*_*E*_ that represents people in *E* moving into *P*_*j*_ because the public system traces the contagion networks. Now, *ϑ*_*P*_ represents people in *P*_*f*+*q*_ = *P*_*f*_ + *P*_*q*_, that do not develop symptoms but move into *P*_*j*_, which models the second strategy. For the third strategy, the identification of symptomatic carriers to be greater than the identification of presymptomatic ones, we proposed that $\vartheta_{P}^{\eta_{\vartheta }}$ (where *η*_*ϑ*_ ∈ [0, 1]) of the population in *L*_*f*+*q*_ (analog to *P*_*f*+*q*_) move into *L*_*j*_.

#### 3.2.5 High symptomatic state (H)

Individuals in *H* have enough viral load to transmit the disease and they develop severe symptoms; thus, the health system rapidly identifies and isolates them, so there is a single compartment *H*_*j*_ in which these individuals stay in mandatory isolation. We assume these individuals can die or recover with time-since-infection probability. It follows that the diffusion system for *H* does not have flows of the form *f* ⇌ *q*/*j*, and therefore random walk matrices will be made up of probabilities of death and recovery. Functions that consider the characteristics of *H*, analogous to those given in the subsection 2.2.4, are defined as follows:

$$\begin{array}{lll} W_{1}^{H}(N_{s},N_{a}) & = & \left\{\begin{array}{ll} 1-\mu g_{\mu }\left(\frac{\ell_{1}(N_{s})}{m}\right)/\underset{w\in \{1,\cdots ,m\}}{\text{max}}g_{\mu }(\frac{w}{m}) & \text{if}\;Q\;\text{holds}\\ 0 & \text{otherwise} \end{array}\right.\\ W_{2}^{H}(N_{s}) & = & \gamma_{H}g_{H}\left(\frac{\ell_{1}(N_{s})}{m}\right)/\underset{w\in \{1,\cdots ,m\}}{\text{max}}g_{H}(\frac{w}{m})\\ W_{3}^{H}(N_{s}) & = & \mu g_{\mu }\left(\frac{\ell_{1}(N_{s})}{m}\right)/\underset{w\in \{1,\cdots ,m\}}{\text{max}}g_{\mu }(\frac{w}{m}) \end{array}$$

where the functions *g*_*μ*_(*x*) and *g*_*H*_(*x*) are parameterized by beta-distribution functions defined as

$$\begin{array}{l} g_{\mu }(x)=\left\{\begin{array}{ll} \frac{x^{a_{\mu }-1}{\left(1-x\right)}^{b_{\mu }-1}}{B(a_{\mu },b_{\mu })} & 0<x<1\\ 0 & \text{otherwise} \end{array}\right.\\ g_{H}(x)=\left\{\begin{array}{cc} \frac{x^{a_{H}-1}{\left(1-x\right)}^{b_{H}-1}}{B(a_{H},b_{H})} & 0<x<1\\ 1 & 1\leq x\\ 0 & \text{otherwise} \end{array}\right. \end{array}$$

Function $W_{3}^{H}\left(N_{s}\right)$ will be used in the following sections to define inside/out flows. In the same way, it must be clear that the income of new high-symptomatic individuals is given by

$$\begin{array}{c} \begin{array}{ccc} H^{1}\left(t+1\right) & = & \left(1-e^{-\phi_{PH}}\right)[P_{f}^{H}\left(t\right)+P_{q}^{H}\left(t\right)+P_{j}^{H}\left(t\right)] \end{array} \end{array}$$

while the total of high-symptomatic individuals is given by

$$\begin{array}{ccc} H\left(t\right) & = & \sum_{i=1}^{m}H^{i}\left(t\right) \end{array}$$

Finally, as an example, the individuals in level *k* ≥ 1 for time-step *t* + 1 would be given by

$$\begin{array}{ccc} H^{k}\left(t+1\right) & = & H^{k-1}\left(t\right)W_{1}^{H}\left(H^{k-1},H^{k}\right)\left(1-W_{2}^{H}\left(H^{k-1}\right)\right) \end{array}$$

which denotes the proportion of individuals in *H*^*k*−1^(*t*) that do not die and do not recover.

#### 3.2.6 Recovery and death states

The disease dynamics finish with recovered individuals from both *L* and *H*, and dead individuals from *H*. These are two separate compartments, and are similar in structure because they acts as sinks.

$$\begin{array}{lll} R(t+1) & = & R(t)+\sum_{i=1}^{m}[L^{i}(t)W_{2}^{L}(L^{i})+H^{i}(t)W_{2}^{H}(L^{i})]\\ D(t+1) & = & D(t)+\sum_{i=1}^{m}H^{i}(t)W_{3}^{H}(H^{i})(1-W_{2}^{H}(H^{i})) \end{array}$$

#### 3.2.7 Environmental reservoir

Assume we have an environmental reservoir of the pathogen constantly renewed by emissions from the infected individuals *I*(*t*). Susceptible individuals *S*(*t*) do have contact with contaminated surfaces at some rate, and then they might get infected. The contact rate is a non-decreasing function of the reservoir size, and without emissions from *I*(*t*), the reservoir would run out. That is the basic idea introduced in [27] to formulate a continuous-time model. We proposed a discrete-time equivalent for that idea defining a threshold from an extreme scenario and setting the behavior of the equation according to that threshold.

In this way, we defined *T*(*t*) as a compartment for the virus concentration in the environment according to the contributions of infectious micro-droplets expelled by *P* and *L* individuals in free circulation. The environmental reservoir can remove the virus from the system by a flow $e^{-\phi_{T}}$. The susceptible individuals could become infected during direct interaction with this reservoir, as exposed in section 3.1.1. We set *T*(*t*) ≤ *k*_*L*_*N*(*t*) because our threshold scenario is the one where all the population becomes infected and symptomatic. Hence, a natural expression for this compartment is given by

$$\begin{array}{c} \begin{array}{ccc} T(t+1) & = & (1-\text{min}[\frac{T(t)}{k_{L}N\left(0\right)},\;1])\left[k_{L}L_{f}\left(t\right)+k_{P}P_{f}\left(t\right)\right]+T\left(t\right)e^{-\phi_{T}} \end{array} \end{array}$$

where *k*_*P*_ is the contribution to the reservoir from *P* and *k*_*L*_ is the contribution of symptomatic carriers to the reservoir. Further, the relation between *k*_*P*_ and *k*_*L*_ turns into how much symptomatic individuals contribute to the environmental reservoir more than presymptomatic. Then, we assumed that the increment is related to the viral load within the carrier. The viral load for a member of *L* could be from 1 to 10 times greater than that of a member of *P* according to [40].

#### 3.2.8 Additional equations for model fitting

When we perform parameter estimation, it is ideal to have the greatest amount of information available to make the model fit. For example, reference intervals for the parameters to be estimated and real data to which the model will fit: time series of the active cases, recovered, and dead (equivalent to node *D*) identified. Note that no explicitly available node is equivalent to said time series in the model; however, it is possible to propose equations that allow the fit without increasing the complexity. For instance, we propose in Eq (4) the following equations for model fitting: *J* as the number of active detected carriers at time *t*, and *R*_*J*_ as the number of detected carriers that have recovered at an specific time.

$$\begin{array}{lll} J(t+1) & = & P_{j}^{L}(t+1)+P_{j}^{H}(t+1)+L_{j}(t+1)+H(t+1)\\ R_{J}(t+1) & = & R_{J}(t)+\sum_{i=1}^{m}[L_{j}^{i}(t)W_{2}^{L}(L_{j}^{i})+H_{j}^{i}(t)W_{2}^{H}(L_{j}^{i})] \end{array}$$ (4)

#### 3.2.9 Simulation of complex phenomena

We identified migration, vaccination, and immunity loss, as complex phenomena with a high impact on the design of public policies. However, we gave up estimating their impact from available data because of the lack of information we would need to validate those estimations and the complexity shift they would cause in the model structure. Then, for the first two, we decided to include them as parameterized model inputs. We modeled such inputs as time series that indicate the impact of the input over the model at each time step. On the other hand, we treated immunity loss as a pulse that allocates a fraction of the recovered individuals into the susceptible ones at the start of the simulation. We based this last decision in the concept of *extension*, which we mentioned in detail in section 2.3. Next, we briefly introduce the parameterizations we proposed to simulate those phenomena.

1. **Migration**: We considered the migratory process as a flow of individuals towards the system. We assumed that symptomatic individuals refrain from migrating, which is a consequence of socially correct behavior. Then, by definition, the migratory flow must consist of susceptible, exposed, and recovered individuals in free circulation. To parameterize time-series generation for migration, we defined seven parameters distributed as follows: two parameters for shape (*a*_*m*_, *b*_*m*_), one parameter for scale (*γ*_*m*_), two parameters for proportion of infectious and recovered migrants $\left(\gamma_{m_{I}},\;\gamma_{m_{R}}\right)$, and two parameters for initial and final time of migration $\left(t_{m_{0}},\;t_{m_{f}}\right)$.
  By using the shape parameters we compute a normal distribution function *f*(*x*) with domain restricted to the interval defined by $A=[t_{m_{0}},\;t_{m_{f}}]$, for instance, *g*(*x*) = *f* ↾_A_ (*x*). The range of *g*(*x*) is then scaled into the interval [0, *γ*_*m*_] defining a new function using the following transformation
  $$h(x)=\left\{\begin{array}{ll} \gamma_{m}\frac{g(x)-\text{min}\;g(x)}{\text{max}\;g(x)-\text{min}\;g(x)} & \text{if}\;x\in A\\ 0 & \text{otherwise} \end{array}\right.$$
  The function *h*(*x*) allows us to achieve a time series when evaluating it at every time step of the simulation. This time series does have the shape of a discrete normal distribution function and a maximum value given by *γ*_*m*_. Finally, we were able to take into account migration slightly modifying the equations for *S*_*q*_, *E*^2^, and *R* as follows. The relation $\gamma_{m_{I}}+\gamma_{m_{R}}\leq 1$ holds.
  $$\begin{array}{lll} S_{f}(t+1) & = & \cdots +(1-\gamma_{m_{I}}-\gamma_{m_{R}})h(t)\;\\ E^{2}(t+1) & = & \cdots \;+\gamma_{m_{I}}h(t)\\ R(t+1) & = & \cdots +\gamma_{m_{R}}h(t) \end{array}$$
2. **Vaccination**: Given the nature of the proposed model, we modeled the vaccination as a flow of people from compartment *S* to compartment *R*. In this way, we were able to treat vaccination as a time series that represents the number of susceptible individuals becoming immune to the disease at each time step, i.e., they acquire the status of recovered. To parameterize this time series, we followed a similar strategy that the one we designed for the recovery/mortality probabilities in diffusion systems (see section 3.1.2). To model vaccination we defined seven parameters as follows: two shape parameters for a beta distribution (*a*_*v*_ and *b*_*v*_), one parameter for vaccine effectiveness (*γ*_*v*_), one parameter for strategy of vaccination (*δ*_*v*_), two parameters for the initial ($t_{v_{0}}$) and the final time ($t_{v_{f}}$) of vaccination program, and a last parameter for the total amount of vaccines (*v*). The total number of effective vaccines would be given by *vγ*_*v*_. We used time parameters to set the domain of the beta distribution function. Finally, we chose a parameter of scale *k*_*v*_ such that
  $$\begin{array}{c} k_{v}\sum_{t=t_{v_{0}}}^{t=t_{v_{f}}}\beta \left(a_{v},\;b_{v},\;t\right)=v\gamma_{v}. \end{array}$$
  being *β*(*a*_*v*_, *b*_*v*_, *t*) the beta distribution function with parameters *a*_*v*_, *b*_*v*_ and domain $\left[t_{v_{0}},t_{v_{f}}\right]$, and *N*_*j*_(*t*) the total amount of detected individuals at time *t* (including the recovered ones that were previously detected). In this way, the vaccination time series would be given by the function
  $$y(t)=\left\{\begin{array}{ll} k_{v}\beta (a_{v},b_{v},t)\frac{S(t)}{N(t)-N_{j}(t)} & \text{if}\;t_{v_{0}}\leq t\leq t_{v_{f}}\\ 0 & \text{otherwise} \end{array}\right.$$
  that we included in the system modifying the equations for *S*_*f*_, *S*_*q*_, and *R* in the following way
  $$\begin{array}{llll} S_{f}(t+1) & = & \cdots & -\text{min}\{\delta_{v}y(t),S_{f}(t)\}\\ S_{q}(t+1) & = & \cdots & -\text{min}\{(1-\delta_{v})y(t),S_{q}(t)\}\\ R(t+1) & = & \cdots & +\text{min}\{\delta_{v}y(t),S_{f}(t)\}+\text{min}\{(1-\delta_{v})y(t),S_{q}(t)\} \end{array}$$
  The meaning of the strategy of vaccination parameter *δ*_*v*_ is the proportion of vaccines to be dosed that requires a displacement of the individuals to some place, which makes them behave as individuals in free circulation, regarding the vaccines to be dosed without exposure of the individuals to infectious sources. Note that proportion *S*(*t*)(*N*(*t*) − *N*_*j*_(*t*))^−1^ models the uncertainty involved when applying vaccines without knowing if the receptor is a real susceptible individual. This expression might be removed according to the particular context of each locality.
3. **Immunity loss**: Reinfection or cross-immunity, is a phenomenon we expected to happen because of the biological properties of the disease alongside its pandemic status. We aimed to model immunity loss by modifying the recovered compartment to flow again into the susceptible one. Hence, we proposed a function *Υ*(*t*) that allocates a small proportion of *R* into *S* (given by immunity-loss parameter *k*_*I*_) at the beginning of the simulation for each extension (see section S1 Appendix for model extensions)
  $$\Upsilon (t)=\left\{\begin{array}{ll} k_{I}\in [0,1] & \text{if}\;t\;\text{is}\;\text{an}\;\text{extension}\;\text{point}\\ 0 & \text{otherwise} \end{array}\right.$$ (5)
  and we modified equations for recovered and susceptible individuals to include *Υ*(*t*) as follows
  $$\begin{array}{llll} S_{f}(t+1) & = & \cdots & +\frac{\lambda_{qf}}{\lambda_{qf}+\lambda_{fq}}\Upsilon (t)R(t)\\ S_{q}(t+1) & = & \cdots & +\frac{\lambda_{fq}}{\lambda_{qf}+\lambda_{fq}}\Upsilon (t)R(t)\\ R(t+1) & = & \cdots & -\Upsilon (t)R(t). \end{array}$$
  One could expect parameter *k*_*I*_ to stay close to 0 for each extension. Therefore, we recommend keeping it as 0 until most of the population recovers ($\frac{R(t)}{N(t)}\geq 0.75$) or the estimation algorithm starts giving poorly feasible parameters (suggesting a high effect of immunity-loss in the dynamics of the disease). We included λ_*qf*_/(λ_*qf*_ + λ_*fq*_) and λ_*fq*_/(λ_*qf*_ + λ_*fq*_) in equations above because we assumed that there is an implicit *f* ⇌ *q* flow within the recovered compartment. Being *R* absorbant, it follows that those terms represent the proportion of individuals in the *f* and *q* sub-states, respectively.

### 3.3 Model implementation for Colombia case

As mentioned in section 2.3, we initially validated the model through UA, SA, and practical IA methodologies described by [32]. During the validation process, we focused on two localities with their initial outbreaks: Colombia and Hubei-China; further information about the description of the results is available at GitHub repository 1. We identified some relevant differences in parameters for social behavior in both localities that suggest Hubei presented a proficient control over the population to avoid disease spread. Also, we highlight that identification of parameters related with tracking of carriers (*η*_*ϑ*_, *ϑ*_*P*_, and *ϑ*_*E*_), quarantine and connectivity parameters (λ_*fq*_, λ_*qf*_, and *ν*) have high importance inside the variance output.

**Fig 5:**
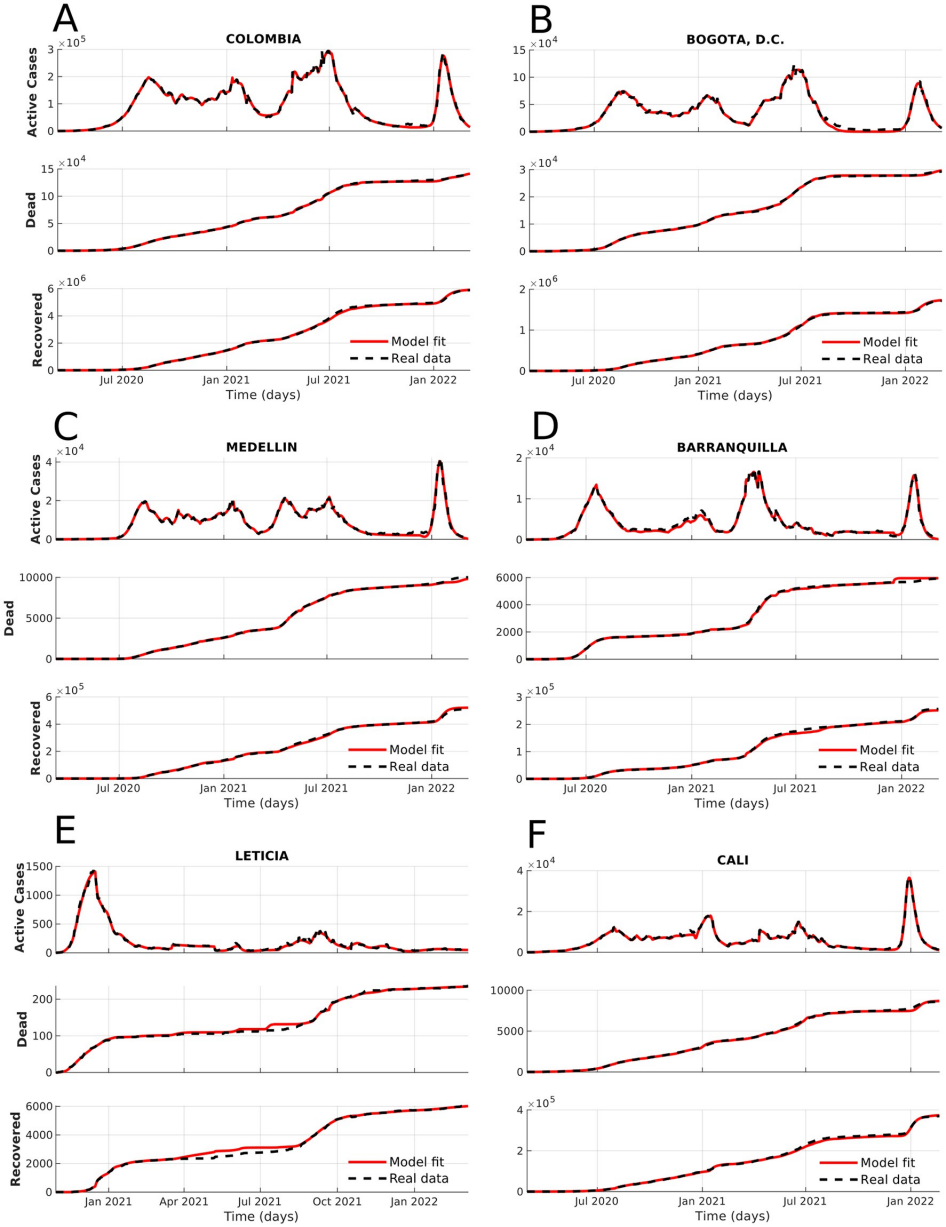
Model fitting for actual, dead, and recovered cases from march 2020 to February 2022. Note that the active cases correspond to the actively infected individuals at a specific moment.

After the corresponding validation (see GitHub repository 1), we implemented the model for different localities in Colombia, where we estimated parameters for all departments of Colombia and their capitals, alongside other specific localities with touristic and economic importance. Thus, we constantly updated 71 localities in Colombia using the algorithm proposed in S1 Appendix from March 2020 until February 2022. In Fig 5, we present six examples of model fitting for some affected localities using three outputs: active cases, dead, and recovered. For example, (A) the entire country and (B) the capital in which we can identify four demarcated outbreaks in actual cases. Also, different localities are big cities, economically important cities, or border cities: (C) Medellín, (D) Barranquilla, (E) Leticia, and (F) Cali. Note that the localities of Bogotá, Medellín, Cali, and Barranquilla hold the whole country trend. Barranquilla has a lower second outbreak compared with the mentioned localities. In contrast, Leticia had one marked peak, followed by small oscillations.

**Fig 6:**
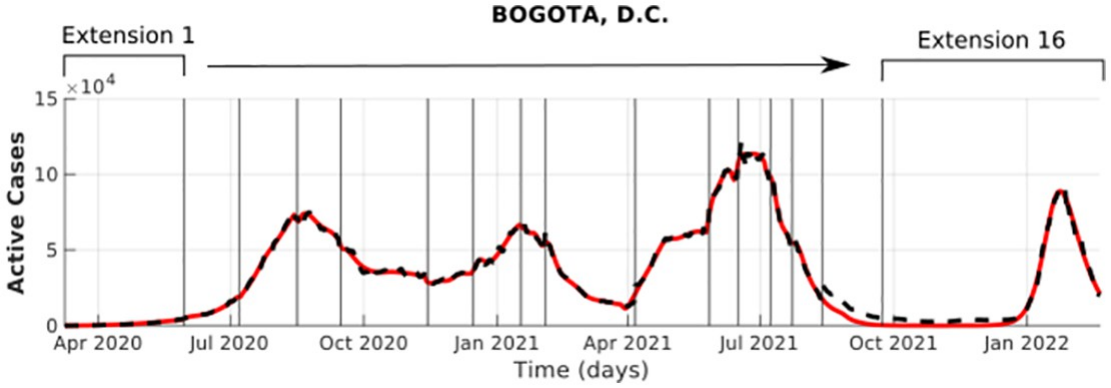
Bogotá fitting for active cases. The vertical lines represent the dates when we stopped using a set of parameters and estimated a new set using the method described in section 2.3 and S1 Appendix. The most representative abrupt changes in the time series are related to social behavior o changes in public policies, e.g., in Bogotá, the first, second and third extensions are related to the gradual relaxation in lockdown policies; the sixth, seventh and eighth extensions are related to increases in interactions and migrations within the population because of vacations and Christmas celebration; the ninth and tenth extensions correspond to an increase in migration because of Easter vacation, and so on.

Each curve fit is composed of different pieces of estimations that we defined as extensions in section 2.3 and estimated them using the algorithm described in S1 Appendix (see Fig 6). For the time series of Colombia, Medellín, Bogotá, Leticia, Cali, and Barranquilla, we divided them into 19, 18, 16, 15, 21, and 27 extensions. For the value and result of each extension, we present validation information available in *.pdf* for each locality in a GitHub repository 2.

#### 3.3.1 Web platform: An evaluation of public policies

We step forward from the design and fitting of the mathematical model to its implementation in a web platform called MathCOVID. It is a platform open to the public that was used as a reference by the epidemiological units in Colombia for all the locations mentioned in this study. Authorities associated with the control implemented the model in the platform to evaluate the effects of public policies on epidemiological indicators (see S2 Appendix). The link to the platform is available in GitHub repository 2.

In the platform, we implemented the parameters that inherently describe control policies as sliders to allow users to evaluate different strategies to compare with the model forecast given by the current parameters. The changes in the parameters are presented in Fig. S4 Appendix as follows:

- Processes of removing the virus from the environment (*ϕ*_*T*_), as cleaning surfaces or closed areas.
- The lockdown process by increasing or decreasing the probabilities of going outside or inside quarantine (λ_*qf*_ and λ_*fq*_). We could define this process as an intermittent strategy (three days of restricted circulation).
- Connectivity level inside populations (*ν*), i.e., can simulate the restriction of the availability of public transport between neighborhoods or localities.
- The number of average interactions between individuals (*z*) as those established in public transport, supermarkets, schools, or at job places.
- The government capacity to track contagion networks (*ϑ*_*E*_), identify asymptomatic and symptomatic carriers (*ϑ*_*P*_ and *η*_*ϑ*_), and finally, the likelihood that people with symptoms will self-isolate (self-care, *η*_*L*_).
- Include migration as an input given by a parameterized time-series that models the addition of new individuals to different compartments, as described in section 2.
- Include vaccination programs as an input given by a parameterized time-series that models the number of vaccines applied to individuals per unit of time. Vaccinated individuals that gained recovered status according to the effectiveness of the vaccine. We refer the reader to section 2 for further reading.

**Fig 7:**
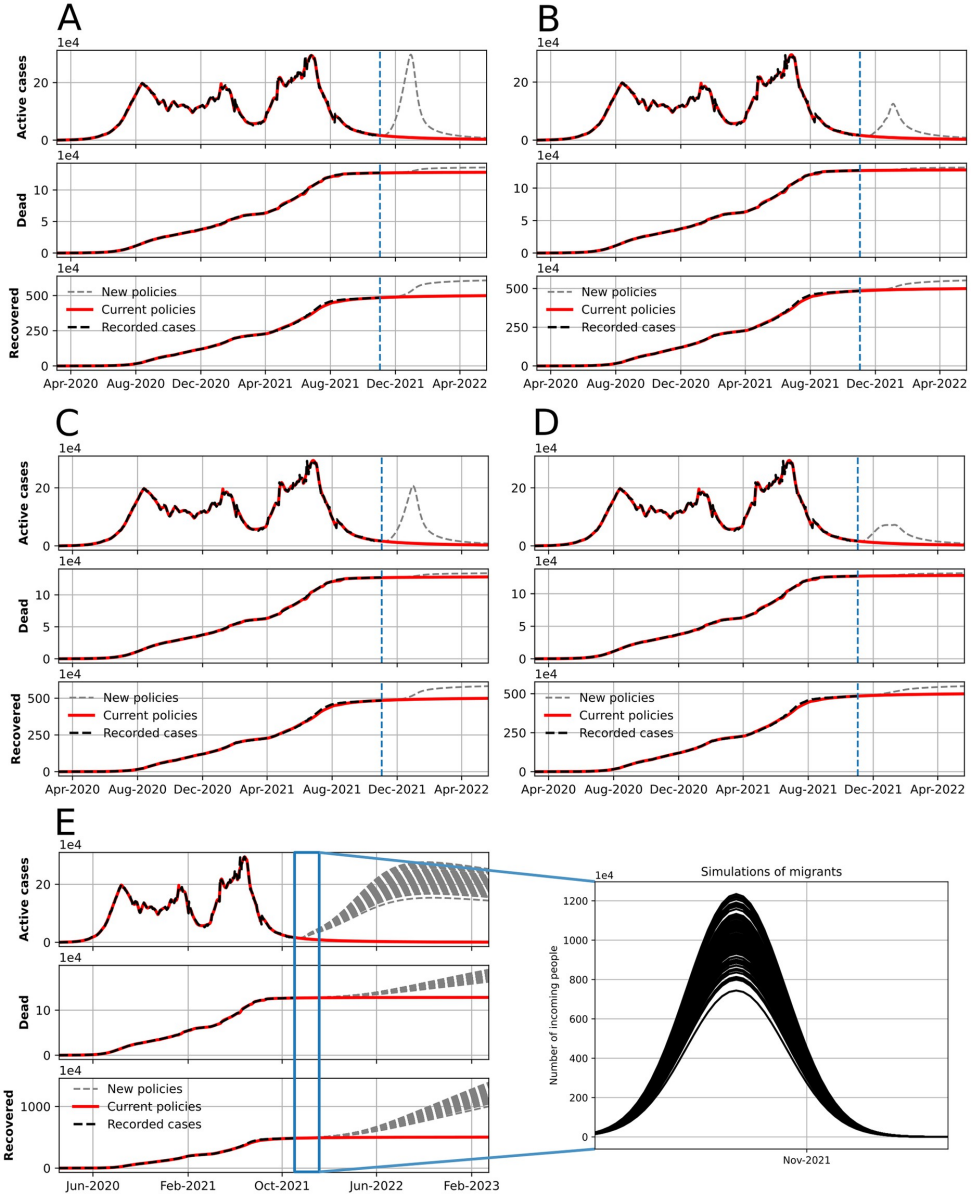
Model simulation using Colombia as example, the blue dotted line shows the limit of model fitting. After that line we present two scenarios, one in which the simulation follows the same trend and other one in which we change some parameters related to public policies. In scenario (A) we present a change in quarantine behavior (λ_*qf*_ from 0.17 to 0.33). In the following figures, we present the original scenario but adding some public policies as (B) intermittent lock-downs as pulse-series, (C) increasing identification programs (λ_*fq*_ in 0.33, *ϑ*_*E*_ from 0.11 to 0.15, and *ϑ*_*P*_ from 0.55 to 0.60), (D) reducing the connectivity inside the country (*ν* from 35 to 150), and (E) a Montecarlo simulation of multiple migration processes hypothesized in November 2021.

**Fig 8:**
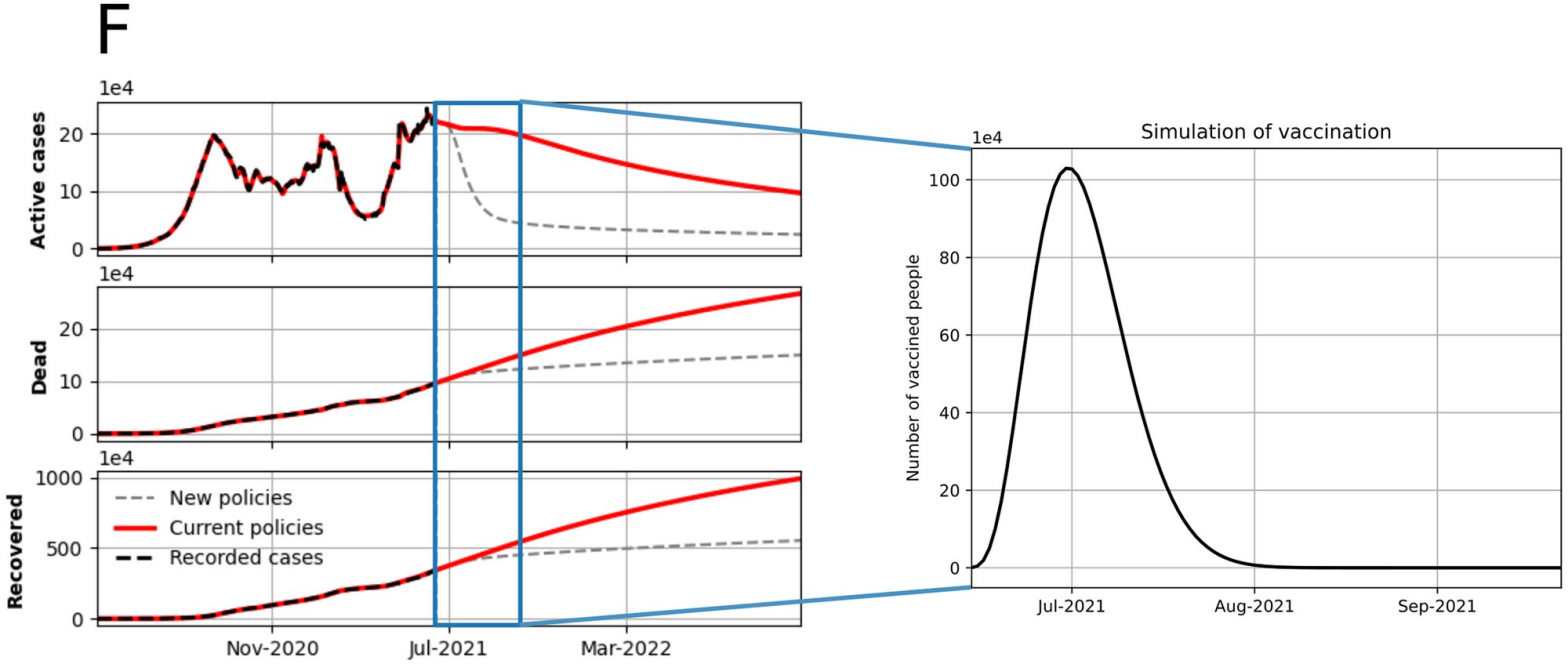
Model simulation using Colombia as an example. The blue line shows the limit of model fitting. After that line, we present two scenarios: one in which the simulation follows the same trend and another one in which we include vaccination policies. Here, we designed the distribution of 20000000 vaccines through an effectiveness of 0.99 and a beta distribution with parameters *a* = 5 and *b* = 20.

Modeling COVID-19 in this way has allowed us to identify possible bad scenarios and simulate outbreaks of COVID-19 in Colombia and some of its localities in the last two years. We present the simulation and the long-term behavior of the system according to model fitting in Figs 7 and 8 for multiple outbreaks in the country. For example:

Scenario (A) is reached by increasing the parameter λ_*qf*_ value (from 0.17 to 0.33) to simulate the social dynamics of the Christmas celebration in Colombia in which the people tend to free circulate triggering the fourth outbreak produced by the appearance of the Omicron strain in Latin America [41, 42]. Note that the curve obtained during this simulation meets the trend of real data in Fig 5, a dynamic behavior also validated in S4 Appendix with Montecarlo simulations. In the following figures, we simulate scenarios with a high flux of people alongside some public policies that could decrease the number of cases of the fourth outbreak. In scenario (B), we present the intermittent lock-down strategy described as pulse-series, in which λ_*fq*_ and λ_*qf*_ are as in S3 Appendix, i.e., people tend to go out every five days and then lockdown.

Scenario (C) in Fig 7, show the simulation of a high flow of people because of Christmas (λ_*fq*_ = 0.33) alongside an increase in the identification programs through parameters *ϑ*_*E*_ (from 0.11 to 0.15) and *ϑ*_*P*_ (from 0.55 to 0.60) that represents an increasing of identification programs effort. Following this strategy, we can obtain a smaller outbreak than the one obtained in scenario (A) and even in the real data presented in Fig 5. On the other hand, in scenario (D), we increased the value of *ν* (from 35 to 150), reducing the movement inside the country, representing a limitation of people from moving to other localities. It has greater impact among the presented scenarios. In scenario (E), we present the case of a considerable migration for a month, in which susceptible people gradually incomes to the country. We can see the effect in the long term, in which detected cases increase considerably after the migration.

We present the vaccination effect in scenario (F) (see Fig 8). We simulated a historical situation of Colombia before implementing mass vaccination (until March 2021) and after it (simulation until July 2021); note that the trend of the simulation with vaccination is similar to the real process carried out in the country during July and August 2021 (see Fig 5).

## 4 Discussion

Since the emergence of the COVID-19 pandemic, some researchers have approached the disease transmission and control study using mathematical models of the *SIR* − *SEIR*-type [3, 4]. However, simplified models exhibit theoretical gaps related to transmission dynamics, e.g., latency or incubation periods, lack of quarantine as a transversal flow, social distancing, and changes in detection strategies [3, 4, 43, 44]; especially for designing and evaluating public policies. Therefore, we developed a model structure and a calibration methodology that remedy these gaps by adapting mathematical expressions for social and epidemiological dynamics. For example, an *f* ⇌ *q*|*j* flow that represents the quarantine and isolation behavior, contagion probabilities that overcome homogeneous dynamics, and diffusion systems for recovery and death dynamics (see section 3.2). All those components together allowed us to model COVID-19 and the effect of public policies.

Our major result is the formulation and validation of a mathematical model developed to simulate public policies using, as a case study, the COVID-19 data. We validated the model alongside parameters and confidence intervals according to different criteria (UA, SA, and practical IA available in GitHub repository 1). The importance of validating the model using the UA, SA and practical IA consist in determining that all proposed parameters are useful and representative of the disease transmission and control in the model. Thus, we implemented the model in several localities with different socio-cultural behavior that share the same data type (infected, recovered, and death cases). For example, Colombia, Bogotá, Medellín, Cali, Barranquilla, Leticia (see section 3.3) and so on presented in GitHub repository 2.

The COVID-19 dynamics in Colombia was similar for Bogotá, Medellín, and Barranquilla; those correspond to large cities with high connectivity between other localities in the country (see Fig 5). For these localities, the first outbreak started after relaxing the quarantine policies. The following outbreaks were related to the increase in contact numbers during Christmas vacations and returning to high-density places (schools, universities, public transport, and jobs). In Cali, there is a similar trend, in which there are multiple outbreaks, but they are lower than the outbreak mostly caused by the Omicron strain, the increase of contacts and the leaving quarantine because of Christmas. For Leticia, the case was completely different. This locality has an explosion of COVID-19 cases at the beginning of the pandemic, then after which there were no new outbreaks, and becoming endemic.

We highlight that the proposed model fits the epidemic dynamics in each study zone; these results suggest progress in fitting transmission models based on real data. For example, as [13] mentioned, some Erlang structures had an issue with identifying and estimating the distribution function related to the diffusion systems, which [45] suggested for future work in the area. We overcame this issue through the estimation method proposed for the complete model, related to the updating process in section 3.3 and the S1 Appendix. We illustrated this process with the study cases of localities in Colombia and China. The calibration of mechanistic models must be constantly updated and recent to capture the disease dynamics and design public policies, specially studying diseases with fast spread as COVID-19 [7].

In section 3.3, we mentioned different policies and their combinations to evaluate and forecast the effect of public policies on an affected population. Through the validation process, we established that the quarantine-related parameters (λ_*fq*_ and λ_*qf*_), carriers identification parameters (*ϑ*_*P*_, *ϑ*_*E*_, and *η*_*ϑ*_), and connectivity inside the population (*ν*) represent the major variance in the output response of the model. Multiple authors discussed this result, concluding that quarantine, regulation in public transport, social distancing, and the use of a mask are valuable strategies to control an epidemic [12, 46, 47]. These policies allowed us to get information about the control conditions to flatten the curve of cases or deaths [8].

Society learned that the zero-COVID policies could suffocate social and economic activities [8]. Thus, it is crucial having interdisciplinary work and expert review during policies design and evaluation using the pros and cons, because people must agree with the policies to shape COVID-19 cases and deaths [8]. We implemented the proposed model on a platform web with more localities to develop comparative studies according to the researcher’s interests. We extended the analysis performed for Colombia to other localities through an interactive tool that decision-makers around Colombia could implement since July 2020. This platform worked as a baseline for designing and analyzing public policies during the most crucial moments in the COVID-19 pandemic in Colombia in 2020–2022. We focused on developing a friendly user interface in which a non-expert mathematician could vary the control parameters in the model and understand how the variations of those parameters could affect the COVID-19 dynamics.

Using Colombia’s data as study case, we presented control scenarios using the information before the Omicron outbreak (see Fig 7); we could identify the different social behaviors and the results in reducing the mentioned outbreak mainly caused by Christmas celebrations. In the first scenario (A), we changed the probability of getting out of quarantine to simulate an out-coming flux due to Christmas celebrations. The scenarios (B) to (D) were created as alternative policies to reduce the number of cases, for example, by implementing intermittent lock-downs, increasing identification programs, or reducing the homogeneous dynamics of the population. The migrant simulation in scenario (E) shows the dynamics of Colombia after receiving a high quantity of migrants into the susceptible population and the final simulation in scenario (F) shows the vaccination process.

Note that we introduced the vaccination and migration as processes in the model as an input instead of a parameter because, for Latin American countries, the vaccine availability was not clear by February 2021. Therefore, we considered it convenient to solve this problem by introducing this process as a time-series input; because it allowed us to evaluate the effect of vaccination in reducing the incidence of COVID-19 in Colombia, as can be seen in Fig 8. Also, we could generate other scenarios that combine vaccination with other public policies associated with quarantine, and the use of masks, among others. Additionally, large-scale vaccination is a process that requires multiple parameters to include and estimate. Hence, developing vaccination as an input reduces the complexity produced by the number of parameters to be estimated, which can generate a large uncertainty in the model output.

Without using a well-known time series to define vaccination input during the estimation process, we could identify some parameters that mitigate the absence of a direct incidence of vaccination. This can be identified in populations with extensions before and after the vaccination started in Colombia. For example, the parameters *ϑ*_*E*_, *z*, *a*_*L*_, and *b*_*L*_ presented changes can be seen in GitHub repository 2 for estimations in Colombia before and after March 2021. Even so, note that the model could capture the dynamics of the disease in Colombia and its localities.

Simulating single or multiple control scenarios offers a useful tool for researchers that involves decision-makers. Thus, we highlight that the proposed model can be adapted according to the natural history of other diseases and countries, i.e., we can use it to model other *SEIR*-type diseases because it is adequate for transmission not associated with COVID-19. For instance, it is possible to create a model without an environmental reservoir and adapt the diffusion systems to describe diseases like influenza or pneumonia. Also, we can add compartments related to vector populations and model the transmission of vector-borne diseases. Thus, it is possible to estimate the under-reported cases through parameter estimation for any target diseases and populations provided there is enough data to fit the model [48].

In the mathematical modeling process, some characteristics prevail: the meaning associated with the parameters and the model fit achieved to the phenomenon [49]. Many mathematical models try to describe, with a degree of generalization or simplification, a real phenomenon in such a way that they can present fitting problems. For example, a classical SIRD model can not adjust to the dead data through a multiobjective optimization problem of fitting positive, dead, and recovered cases of China [50]. Therefore, the complexity of describing the real process must be evaluated, as well as other modeler objectives as the development of control measures, such as public policies associated with epidemiological events. In our case, we considered incorporating different public policies and the information available for this disease in Colombia (identified cases, recovered cases, and deaths). We highlight that because of the availability and quality of the data registered for COVID-19 in Colombia, it was possible to develop a detailed model, presented in section 2.2.

The complexity of the proposed model must be linked to the data availability and the main interests of the researcher. For example, we focused on the behavior of the general population to develop and validate a method for modeling public policies. Even so, because of the age-related severity in COVID-19 [51, 52], the mathematical structure proposed in this paper could be implemented to model meta-population as age-divided groups by defining and linking multiple models for the age-structures [53]. Also, if data by age groups is available, parameter estimation for the age groups could be carried out as future work in the area. We encourage the development of a formal study of the computational time required for the model calibration and validation. Even so, the time taken for model fitting satisfied the necessities required by the decision-makers in Colombia. Finally, note that in this paper, we focused more on identifying the effect of public policies than developing and studying *R*_0_ and *R*_*t*_ measures, considering the difficulties exposed in [54].

## 5 Conclusion

We have shown the potential to model *SEIR*-type dynamics using a structure that combines contact probabilities for heterogeneous populations, a quarantine, and free circulation flows from Erlang model theory, and diffusion systems that describe death and recovery dynamics. This potential was evidenced by fitting the proposed discrete model in more than 70 localities in Colombia for the first two years of the COVID-19 pandemic (see GitHub repository 1 and GitHub repository 2).

Also, we gave a step further by implementing the model into a web platform as a tool for helping the decision-makers to evaluate the effects or to design public policies such as lock-downs, social distancing, identification of contagion networks, migration, vaccination, and connectivity among populations. Local governments and institutions, such as the *Universidad EAFIT*, implemented this platform to identify when and how often to reintegrate into their professional activities and reduce its effects on the active cases. Also, some economic activities in the country, such as the Coffee Growing Area, implemented the model in 2020 to identify the epidemic dynamics and evaluate the control strategies effects. Finally, we observe that future researchers could implement the proposed method and model structure for other countries and diseases.

## Supporting information

S1 Appendix Estimation and validation of parameters for the extension process. (PDF)

S2 Appendix Model implementation in a web platform (MathCOVID) for the usage of decision-makers in Colombia. (PDF)

S3 Appendix Intermittent quarantine designed as multiple pulse signals depending on time. (PDF)

S4 Appendix Montecarlo simulation for Colombia. (PDF)

## References

[1] Kim S, Lee J, Jung E. Mathematical model of transmission dynamics and optimal control strategies for 2009 A/H1N1 influenza in the Republic of Korea. Journal of Theoretical Biology. 2017;412:74–85. doi: 10.1016/j.jtbi.2016.09.025 27769686

[2] Zhou Y, Ma Z, Brauer F. A discrete epidemic model for SARS transmission and control in China. Mathematical and Computer Modelling. 2004;40(13):1491–1506. doi: 10.1016/j.mcm.2005.01.007 32288200 PMC7135158

[3] Catano-Lopez A, Rojas-Diaz D. Modelos discretos de transmisión de COVID-19 y publicaciones preeliminares en la ciencia: una búsqueda sistematizada. Scielo pre-prints. 2020. 10.1590/scielopreprints.1076

[4] Anastassopoulou C, Russo L, Tsakris A, Siettos C. Data-based analysis, modelling and forecasting of the COVID-19 outbreak. PLOS ONE. 2020;15(3):e0230405. doi: 10.1371/journal.pone.0230405 32231374 PMC7108749

[5] Goldsztejn U, Schwartzman D, Nehorai A. Public policy and economic dynamics of COVID-19 spread: A mathematical modeling study. PLOS ONE. 2020;15(12):e0244174. doi: 10.1371/journal.pone.0244174 33351835 PMC7755180

[6] Silva CJ, Cruz C, Torres DFM, Muñuzuri AP, Carballosa A, Area I, et al. Optimal control of the COVID-19 pandemic: controlled sanitary deconfinement in Portugal. Scientific Reports. 2021;11(1). doi: 10.1038/s41598-021-83075-6 33568716 PMC7876047

[7] Adiga A, Dubhashi D, Lewis B, Marathe M, Venkatramanan S, Vullikanti A. Mathematical Models for COVID-19 Pandemic: A Comparative Analysis. Journal of the Indian Institute of Science. 2020;100(4):793–807. doi: 10.1007/s41745-020-00200-6 33144763 PMC7596173

[8] Su Z. Rigorous Policy-Making Amid COVID-19 and Beyond: Literature Review and Critical Insights. International Journal of Environmental Research and Public Health. 2021;18(23):12447. doi: 10.3390/ijerph182312447 34886171 PMC8657108

[9] Chretien JP, George D, Shaman J, Chitale RA, McKenzie FE. Influenza Forecasting in Human Populations: A Scoping Review. PLoS ONE. 2014;9(4):e94130. doi: 10.1371/journal.pone.0094130 24714027 PMC3979760

[10] Funk S, Camacho A, Kucharski AJ, Eggo RM, Edmunds WJ. Real-time forecasting of infectious disease dynamics with a stochastic semi-mechanistic model. Epidemics. 2018;22:56–61. doi: 10.1016/j.epidem.2016.11.003 28038870 PMC5871642

[11] Yamana TK, Kandula S, Shaman J. Individual versus superensemble forecasts of seasonal influenza outbreaks in the United States. PLOS Computational Biology. 2017;13(11):e1005801. doi: 10.1371/journal.pcbi.1005801 29107987 PMC5690687

[12] Riyapan P, Shuaib SE, Intarasit A. A Mathematical Model of COVID-19 Pandemic: A Case Study of Bangkok, Thailand. Computational and Mathematical Methods in Medicine. 2021;2021:1–11. doi: 10.1155/2021/6664483 33815565 PMC8010525

[13] Getz WM, Dougherty ER. Discrete stochastic analogs of Erlang epidemic models. Journal of Biological Dynamics. 2017;12(1):16–38. doi: 10.1080/17513758.2017.1401677 PMC6120589 29157162

[14] Köhler-Rieper F, Röhl CHF, De Micheli E. A novel deterministic forecast model for COVID-19 epidemic based on a single ordinary integro-differential equation. European Physical Journal Plus. 2020;135(7):19. doi: 10.1140/epjp/s13360-020-00608-0 32834915 PMC7381419

[15] Keimer A, Pflug L. Modeling infectious diseases using integro-differential equations: Optimal control strategies for policy decisions and Applications in COVID-19; 2020. Available from: http://rgdoi.net/10.13140/RG.2.2.10845.44000.

[16] Dell’Anna L. Solvable delay model for epidemic spreading: the case of Covid-19 in Italy. Scientific Reports. 2020;10(1):1–10. doi: 10.1038/s41598-020-72529-y 32978440 PMC7519166

[17] Logeswari K, Ravichandran C, Nisar KS. Mathematical model for spreading of COVID-19 virus with the Mittag-Leffler kernel. Numerical Methods for Partial Differential Equations. 2020;77(1). doi: 10.1002/num.22652 33362342 PMC7753447

[18] Viguerie A, Lorenzo G, Auricchio F, Baroli D, Hughes TJR, Patton A, et al. Simulating the spread of covid-19 via a spatially-resolved susceptible-exposed-infected-recovered-deceased (seird) model with heterogeneous diffusion. Applied Mathematics Letters. 2020;111(1):9. 10.1016/j.aml.2020.106617 PMC7361091 32834475

[19] Balabdaoui F, Mohr D. Age-stratified model of the COVID-19 epidemic to analyze the impact of relaxing lockdown measures: nowcasting and forecasting for Switzerland. medRxiv. 2020; p. 1–19. doi: 10.1101/2020.05.08.20095059

[20] Martcheva M. An Introduction to Mathematical Epidemiology. Springer {US}; 2015. Available from: 10.1007/978-1-4899-7612-3.

[21] World Health Organization. Coronavirus disease 2019 (COVID-19), Situation Report − 93; 2020. https://www.who.int/emergencies/diseases/novel-coronavirus-2019/situation-reports.

[22] Liu Y, Ning Z, Chen Y, Guo M, Liu Y, Gali NK, et al. Aerodynamic Characteristics and RNA Concentration of SARS-CoV-2 Aerosol in Wuhan Hospitals during COVID-19 Outbreak. BioRxiv. 2020.

[23] Centers for Disease Control and Prevention. How COVID-19 Spreads; 2020. https://www.cdc.gov/coronavirus/2019-ncov/about/transmission.html. 34009769

[24] Fink JB, Ehrmann S, Li J, Dailey P, McKiernan P, Darquenne C, et al. Reducing Aerosol-Related Risk of Transmission in the Era of COVID-19: An Interim Guidance Endorsed by the International Society of Aerosols in Medicine. Journal of Aerosol Medicine and Pulmonary Drug Delivery. 2020. doi: 10.1089/jamp.2020.1615 32783675 PMC7757542

[25] European Centre for Disease Prevention and Control. Transmission of COVID-19; 2020. https://www.ecdc.europa.eu/en/covid-19/latest-evidence/transmission.

[26] Oran DP, Topol EJ. Prevalence of Asymptomatic SARS-CoV-2 Infection. Annals of Internal Medicine. 2020;173(5):362–367. doi: 10.7326/M20-3012 32491919 PMC7281624

[27] Yang C, Wang J. A mathematical model for the novel coronavirus epidemic in Wuhan, China. Mathematical Biosciences and Engineering. 2020;17(3):2708–2724. doi: 10.3934/mbe.2020148 32233562 PMC7376496

[28] Catano-Lopez A, Rojas-Diaz D, Lizarralde DP, Puerta Yepes ME. Discrete Models in Epidemiology: New Contagion Probability Functions Based on Real Data Behavior. Bulletin of Mathematical Biology. 2022;84(127):1–23 2022. doi: 10.1007/s11538-022-01076-6 36138179 PMC9510274

[29] He X, Lau EHY, Wu P, Deng X, Wang J, Hao X, et al. Temporal dynamics in viral shedding and transmissibility of COVID-19. Nature Medicine. 2020;26(5):672–675. doi: 10.1038/s41591-020-0869-5 32296168

[30] Champredon D, Dushoff J, Earn DJD. Equivalence of the Erlang-Distributed SEIR Epidemic Model and the Renewal Equation. SIAM Journal on Applied Mathematics. 2018;78(6):3258–3278. doi: 10.1137/18M1186411

[31] Rojas-Díaz, Daniel and Vélez-Sánchez, Carlos Mario. drojasd/GSUA-CSB: GSUA-CSB v1.0; 2019. Available from: https://zenodo.org/record/3383316 [cited 2019-09].

[32] Lizarralde-Bejarano DP, Rojas-Díaz D, Arboleda-Sánchez S, Puerta-Yepes ME. Sensitivity, uncertainty and identifiability analyses to define a dengue transmission model with real data of an endemic municipality of Colombia. PLOS ONE. 2020;15(3):e0229668. doi: 10.1371/journal.pone.0229668 32160217 PMC7065780

[33] Catano-Lopez A, Rojas-Diaz D. Chimera Model For Covid19; 2021. https://github.com/alexacl95/ChimeraModelForCovid19.

[34] Hernandez-Ceron N, Feng Z, Castillo-Chavez C. Discrete Epidemic Models with Arbitrary Stage Distributions and Applications to Disease Control. Bulletin of Mathematical Biology. 2013;75(10):1716–1746. doi: 10.1007/s11538-013-9866-x 23797790 PMC4002294

[35] Tang W, Cao Z, Han M, Wang Z, Chen J, Sun W, et al. Hydroxychloroquine in patients with mainly mild to moderate coronavirus disease 2019: open label, randomised controlled trial. BMJ. 2020; p. m1849. doi: 10.1136/bmj.m1849 32409561 PMC7221473

[36] Dong E, Du H, Gardner L. An interactive web-based dashboard to track COVID-19 in real time. The Lancet Infectious Diseases. 2020;20(5):533–534. doi: 10.1016/S1473-3099(20)30120-1 32087114 PMC7159018

[37] Cancino A, Gajardo P, Lecaros R, Muñoz C, Ramírez H, Ortega J. Report #1: estimation of maximal critical health facilities demand for COVID-19 outbreak in Santiago, Chile; 2020. Available from: http://www.cmm.uchile.cl/?p=37663.

[38] Carraturo F, Giudice CD, Morelli M, Cerullo V, Libralato G, Galdiero E, et al. Persistence of SARS-CoV-2 in the environment and COVID-19 transmission risk from environmental matrices and surfaces. Environmental Pollution. 2020;265:115010. doi: 10.1016/j.envpol.2020.115010 32570023 PMC7280109

[39] jie Guan W, yi Ni Z, Hu Y, hua Liang W, quan Ou C, xing He J, et al. Clinical Characteristics of Coronavirus Disease 2019 in China. New England Journal of Medicine. 2020;382(18):1708–1720. doi: 10.1056/NEJMoa2002032 32109013 PMC7092819

[40] Stadnytskyi V, Bax CE, Bax A, Anfinrud P. The airborne lifetime of small speech droplets and their potential importance in SARS-CoV-2 transmission. Proceedings of the National Academy of Sciences. 2020;117(22):11875–11877. doi: 10.1073/pnas.2006874117 32404416 PMC7275719

[41] Du X, Tang H, Gao L, Wu Z, Meng F, Yan R, et al. Omicron adopts a different strategy from Delta and other variants to adapt to host. Signal Transduction and Targeted Therapy. 2022;7(1). doi: 10.1038/s41392-022-00903-5 35145066 PMC8830988

[42] Taylor L. Covid-19: Omicron drives weekly record high in global infections. BMJ. 2022; p. o66. doi: 10.1136/bmj.o66 35017144

[43] Grant A. Dynamics of COVID-19 epidemics: SEIR models underestimate peak infection rates and overestimate epidemic duration. medRxiv. 2020. doi: 10.1101/2020.04.02.20050674

[44] Comunian A, Gaburro R, Giudici M. Inversion of a SIR-based model: A critical analysis about the application to COVID-19 epidemic. Physica D: Nonlinear Phenomena. 2020;413:132674. doi: 10.1016/j.physd.2020.132674 32834252 PMC7419377

[45] Champredon D, Dushoff J. Intrinsic and realized generation intervals in infectious-disease transmission. Proceedings of the Royal Society B: Biological Sciences. 2015;282(1821):20152026. doi: 10.1098/rspb.2015.2026 26674948 PMC4707754

[46] Arellana J, Márquez L, Cantillo V. COVID-19 Outbreak in Colombia: An Analysis of Its Impacts on Transport Systems. Journal of Advanced Transportation. 2020;2020:1–16. doi: 10.1155/2020/8867316

[47] Yang HM, Junior LPL, Castro FFM, Yang AC. Mathematical modeling of the transmission of SARS-CoV-2—Evaluating the impact of isolation in São Paulo State (Brazil) and lockdown in Spain associated with protective measures on the epidemic of CoViD-19. PLOS ONE. 2021;16(6):e0252271. doi: 10.1371/journal.pone.0252271 34129608 PMC8205178

[48] Giordano G, Blanchini F, Bruno R, Colaneri P, Filippo AD, Matteo AD, et al. Modelling the COVID-19 epidemic and implementation of population-wide interventions in Italy. Nature Medicine. 2020. doi: 10.1038/s41591-020-0883-7 32322102 PMC7175834

[49] Huppert A, Katriel G. Mathematical modelling and prediction in infectious disease epidemiology. Clinical Microbiology and Infection. 2013;19(11):999–1005. doi: 10.1111/1469-0691.12308 24266045

[50] Sen D, Sen D. Use of a Modified SIRD Model to Analyze COVID-19 Data. Industrial & Engineering Chemistry Research. 2021;60(11):4251–4260. doi: 10.1021/acs.iecr.0c04754 37556235

[51] Roy S, Ghosh P. Factors affecting COVID-19 infected and death rates inform lockdown-related policymaking. PLOS ONE. 2020;15(10):e0241165. doi: 10.1371/journal.pone.0241165 33095811 PMC7584177

[52] Banerjee D. The impact of Covid-19 pandemic on elderly mental health. International Journal of Geriatric Psychiatry. 2020;35(12):1466–1467. doi: 10.1002/gps.5320 32364283 PMC7267435

[53] Bichara D, Kang Y, Castillo-Chavez C, Horan R, Perrings C. SIS and SIR Epidemic Models Under Virtual Dispersal. Bulletin of Mathematical Biology. 2015;77(11):2004–2034. doi: 10.1007/s11538-015-0113-5 26489419 PMC4749480

[54] Li J, Blakeley D, Smith RJ. The Failure of *R*_0_. Computational and Mathematical Methods in Medicine. 2011;2011:1–17. doi: 10.1155/2011/527610 21860658 PMC3157160

