# Supplementary material for: A discrete model for the evaluation of public policies: The case of Colombia during the COVID-19 pandemic
Source: PLoS One. 2023 Feb 14;18(2):e0275546. doi: 10.1371/journal.pone.0275546 (PMC9928135; doi:10.1371/journal.pone.0275546)
Supplement: S1 Appendix — (PDF) [file pone.0275546.s001.pdf]

## S1: Estimation and validation of parameters for the extension process

Periodically, we downloaded the real data and updated the model for the localities (see section 1.3 and *GitHub repository 2*). Thus, after a first parameter estimation (first extension), we had information segments for different periods for each locality (posterior extensions). In Fig S1, we present the main workflow for fitting the first extension and posterior extensions.

After downloading real data, we divide the workflow into two ways. (i) For new locations we identify abrupt changes in real data by determining the number of extensions to split the time series. (ii) For processed locations, we add new data segments and determine whether to update the locality (generate a new extension) or to grow the last extension. Then, for each case (new and processed localities), we perform the parameter estimation for each extension. Finally, the parameters obtained from the estimation process for each extension and locality are saved into a structure; thus, we have a record of parameters for each segment with abrupt changes.

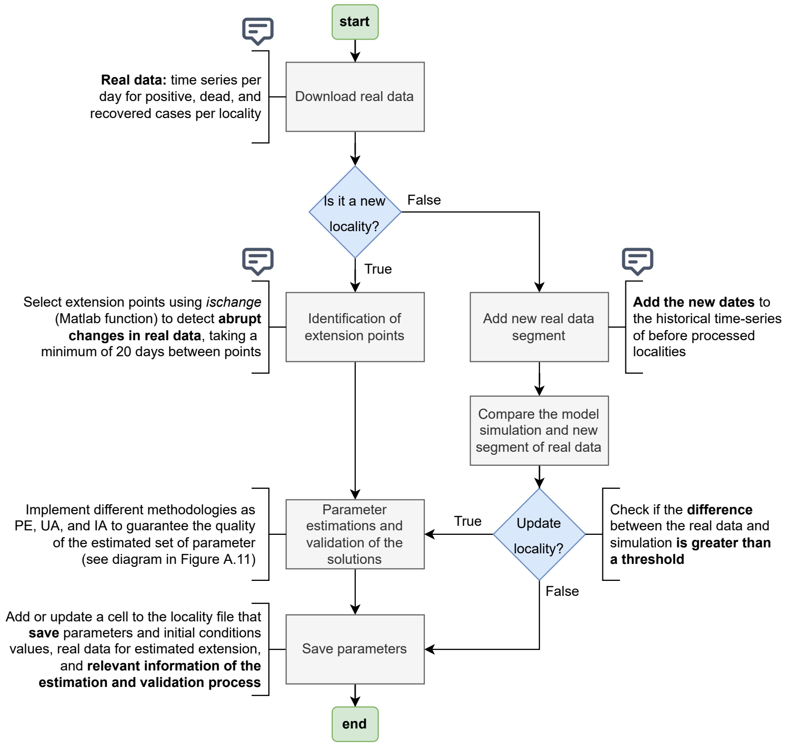


**Fig S1.** Main flowchart that describes the update of the localities through parameter estimation (PE), validation of estimation (using SA and UA), and save the obtained information.

We address the process of validation for parameter estimations that we show in Fig S2. Internally, the algorithm defines some fixed parameter and initial conditions values for

each locality and feasible estimation intervals for parameters and initial conditions. Also, it has a list of parameters that could be fixed in extra rounds of parameter estimations. First, the algorithm performs $N_p$ parameter estimations (initially set on 1000) with their corresponding residuals. We set the first threshold in which the algorithm selects those estimations that are less than $n_1$ times the best cost function value (initially set on 30%). If only one curve meets this criterion, the algorithm will perform another round of parameter estimations but increase the estimations number by adding $N_n$ (set on 1000). In the other case, the algorithm estimates an identifiability index and parametric confidence intervals for each parameter; both methodologies are implemented in the GSUA_CSB toolbox. If the number of estimations selected with $n_1$ criteria is less than $t_1$, the algorithm selects one parameter to fix (following the identifiability index) and performs an extra round of parameter estimations. On the other hand, if the number of estimations selected is greater than $t_1$, the algorithm estimates the difference through residuals between curves obtained with the median of the parameters and the best-estimated curve. If the difference between both curves is lower than 30%, then the algorithm performs a heuristic method to estimate confidence intervals (see Sub-contour Box in [1]). Then, the algorithm completes saving the set of parameters of the best estimation.

Where the difference between the median and best-estimated curve is greater than 30% and there are less than $t_2$ curves, the algorithm generates a $N_S$ montecarlo simulation using parametric intervals. If these simulations' residuals are less than $n_2$ times the best residual, the algorithm will reduce the $n_1$ value by 2%. In this case, the algorithm will select again the residuals that are less than the new threshold $N_1$; it could improve the median intervals and fit.

# References


1. Rojas-Diaz D, Catano-Lopez A, Velez-Sanchez CM. A novel algorithm for confidence sub-contour box estimation: an alternative to traditional confidence intervals; 2019.

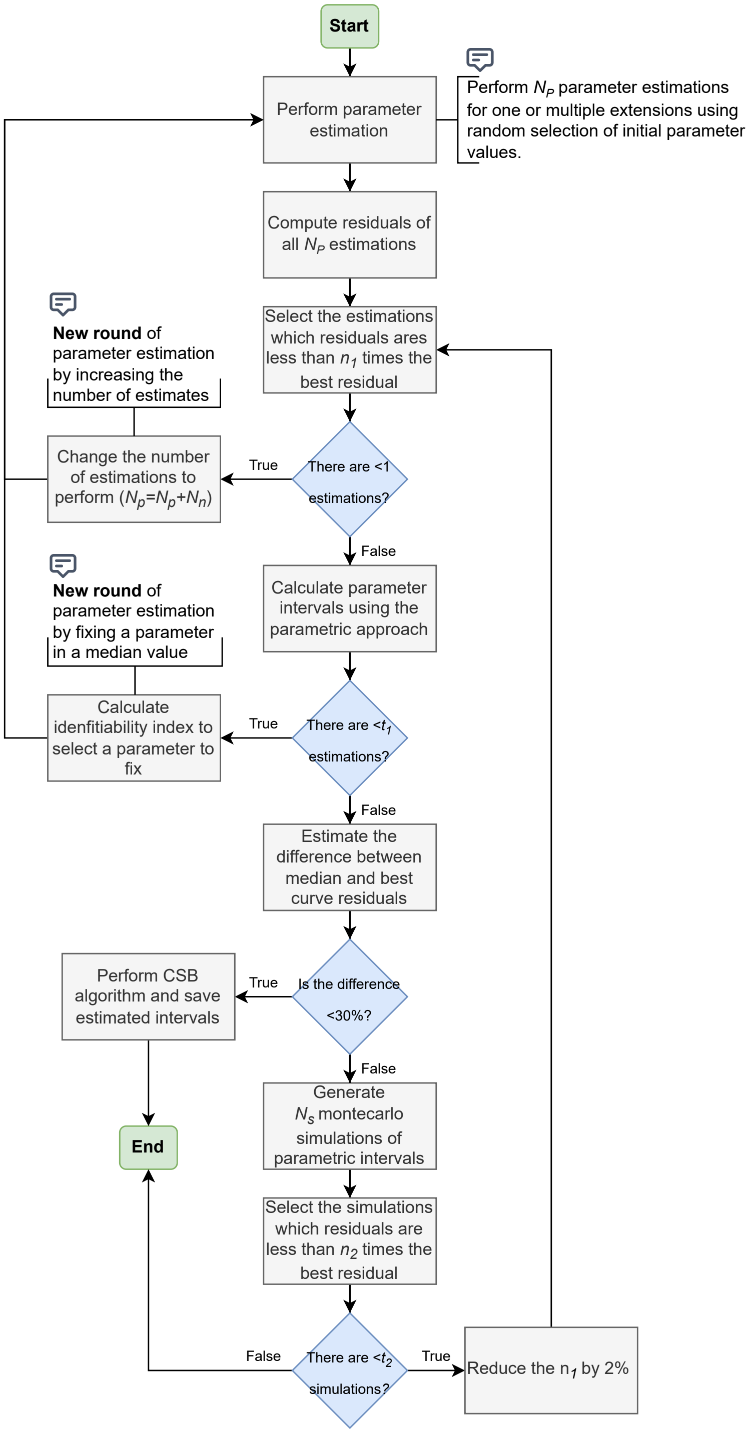


**Fig S2.** Flowchart for parameter estimation and it validation for new and processed localities.
