# Supplementary material for: A discrete model for the evaluation of public policies: The case of Colombia during the COVID-19 pandemic
Source: PLoS One. 2023 Feb 14;18(2):e0275546. doi: 10.1371/journal.pone.0275546 (PMC9928135; doi:10.1371/journal.pone.0275546)
Supplement: S2 Appendix — (PDF) [file pone.0275546.s002.pdf]

S2: Model implementation in a Web Platform (MathCOVID) for the usage of decision-makers in Colombia

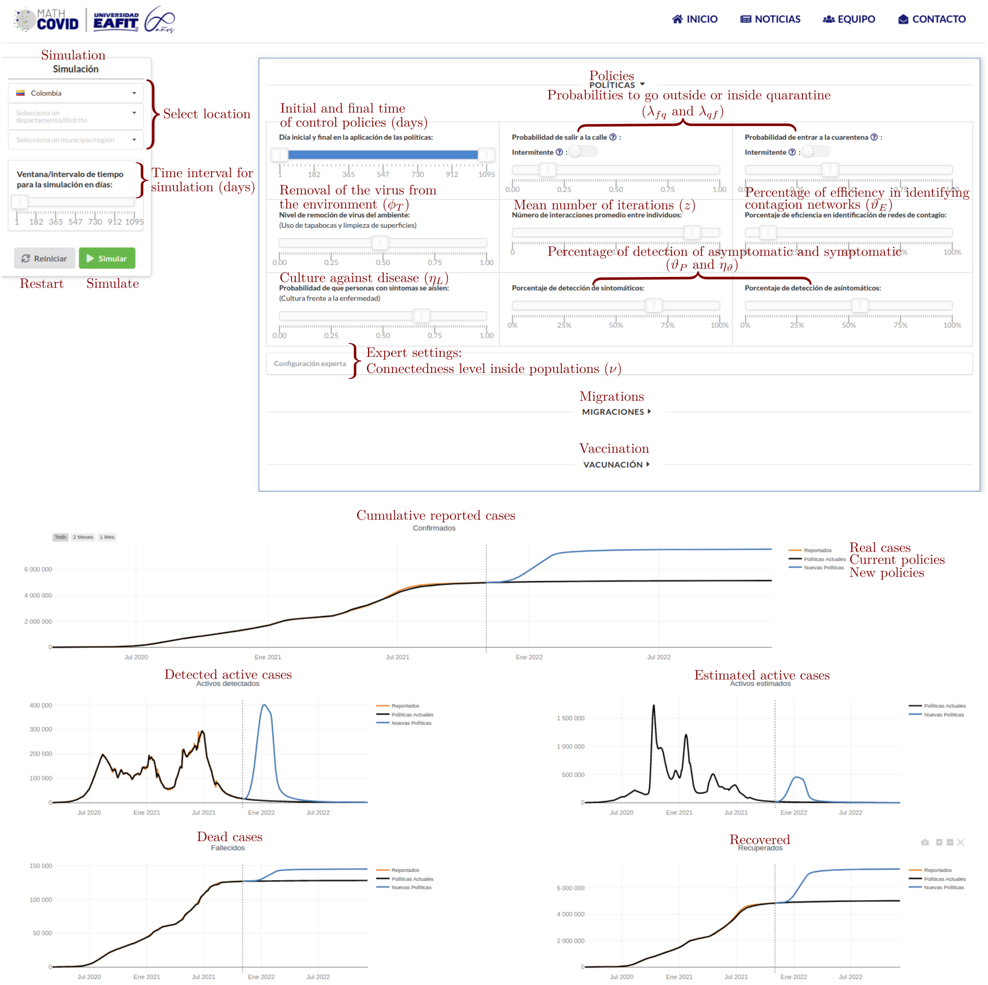

Fig S3. Model implementation in a Web Platform (MathCOVID) for the usage of decision-makers in Colombia. Text in red is an explanation of each model component related to control parameters. The link to the platform is available in *GitHub repository 2*.
