# Supplementary material for: A discrete model for the evaluation of public policies: The case of Colombia during the COVID-19 pandemic
Source: PLoS One. 2023 Feb 14;18(2):e0275546. doi: 10.1371/journal.pone.0275546 (PMC9928135; doi:10.1371/journal.pone.0275546)
Supplement: S3 Appendix — (PDF) [file pone.0275546.s003.pdf]

S3: Intermittent quarantine designed as multiple pulse signal depending on time

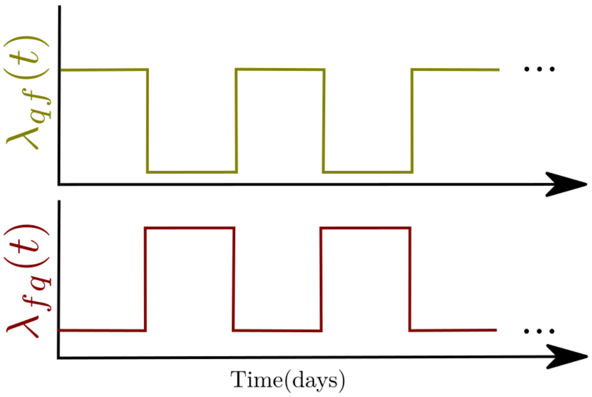

Fig S4. Intermittent quarantine is designed as a multiple pulse signal depending on time. For Fig 7, the first parameter values are $\lambda_{qf} = 0.05$ and $\lambda_{fq} = 0.5$; the second parameter values are $\lambda_{qf} = 0.33$ and $\lambda_{fq} = 0.15$. The frequency of the changes between the first and second values are five days.
