# Supplementary material for: A discrete model for the evaluation of public policies: The case of Colombia during the COVID-19 pandemic
Source: PLoS One. 2023 Feb 14;18(2):e0275546. doi: 10.1371/journal.pone.0275546 (PMC9928135; doi:10.1371/journal.pone.0275546)
Supplement: S4 Appendix — (PDF) [file pone.0275546.s004.pdf]

S4: Montecarlo simulations

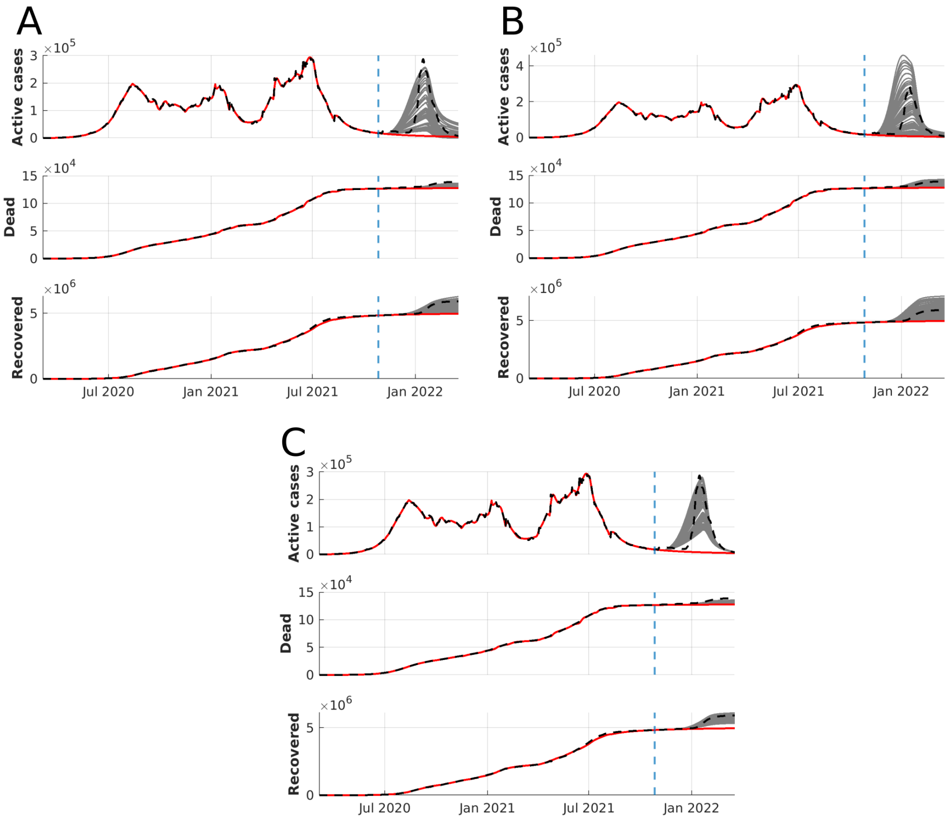

Fig S5. Montecarlo simulation using Colombia as an example for design multiple control policies. Note that (i) the black dotted line is the real data. (ii) The blue dotted one represents how far we adjusted the model. (iii) The red line is the simulation using the parameters estimated until the blue dotted line. (iv) The gray lines are model simulations fixing almost all parameters and performing a pulse signal as described in supplementary Fig S4. We varied for (A) $\lambda_{fq} = [0.2, 0.3]$ in November and $\lambda_{fq} = [0.01, 0.2]$ in January, For (B) $\lambda_{qf} = [0.2, 0.4]$ in November and $\lambda_{qf} = [0.4, 0.5]$ in January, and (C) $\lambda_{fq} = 0.2$ and $z = [25, 30]$ in November, and $\lambda_{fq} = 0.5$ and $z = [15, 20]$ in January. During the simulation of these new policies and strategies, we can identify that the model has long-term dynamics that contain the real behavior that occurred after the blue line. Within this paradigm, the interest is to identify if it is possible to capture the general dynamics of a phenomenon.
